# Dynamic adsorption behavior and film-forming stabilization mechanism of brown rice protein isolate at the oil-water interface: Effects of short-term germination treatment

**DOI:** 10.1016/j.crfs.2026.101615

**Published:** 2026-11-02

**Authors:** Zhuangpeng Wang, Xinwen Huang, Yu Zhao, Haiping Liang, Kun Wang, Leiyan Wu

**Affiliations:** a College of Agriculture, Jiangxi Agricultural University, Nanchang, 330045, China; b Ministry of Education and Jiangxi Key Laboratory of Crop Physiology, Ecology and Genetic Breeding, Jiangxi Agricultural University, Nanchang, 330045, China; c College of Food Science and Engineering, Jiangxi Agricultural University, Nanchang, 330045, China

**Keywords:** Brown rice protein isolate, Germination, Protein structural remodeling, Interfacial adsorption kinetics, Interfacial dilatational rheology, Emulsifying properties

## Abstract

Protein interfacial adsorption is fundamental to the formation and stabilization of emulsions. However, the limited interfacial adsorption performance of brown rice protein isolate (BRPI) restricts its application as a plant-based emulsifier. This study investigated how germination modifies BRPI structure and how these changes relate to interfacial adsorption, film mechanics, and emulsifying performance. Untreated, soaked, and germinated BRPI were compared through structural characterization, interfacial adsorption kinetics, dilatational rheology, and emulsion evaluation. Soaking alone induced slight aggregation and reduced emulsifying activity, whereas subsequent germination promoted partial protein hydrolysis and conformational loosening, accompanied by smaller particle sizes and increased solubility, conformational flexibility, and surface hydrophobicity. These changes were associated with faster interfacial adsorption and the formation of stronger, elasticity-dominated interfacial films. Among the investigated treatments, 24 h germination yielded the highest emulsifying activity index and emulsifying stability index, increasing from 2.61 to 4.49 m^2^/g and from 39.15 to 191.49 min, respectively, compared with untreated BRPI, and produced finer, more uniformly dispersed emulsion droplets. Correlation analysis showed that both emulsifying indices were positively associated with diffusion, penetration, and rearrangement rate constants, interfacial dilatational elastic modulus, and the *E–π* slope. These findings support a mechanism in which germination-induced structural remodeling improves protein adsorption and interfacial film elasticity, jointly facilitating emulsion formation and stabilization. Controlled germination therefore represents a promising strategy for enhancing the functionality of BRPI as a plant-based functional emulsifier.

## Highlights

• Revealed the specific pathway of BRPI degradation by hydrolases during germination.
• Elucidated how germinated BRPI adsorbs at the O/W interface to stabilize emulsions.
• Established a correlation model between structure, interface, and emulsification.

## Graphical abstract

**Figure:**
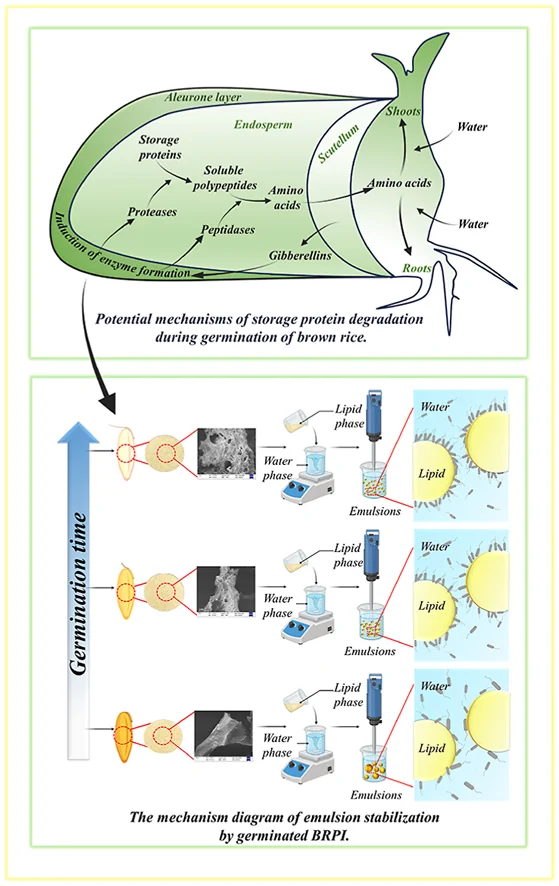

## 1 Introduction

To address the growing protein resource scarcity driven by intensifying global climate change and population growth, as well as the rising consumer demand for plant-based foods, plant-derived proteins have emerged as a research hotspot (W. Chen et al., 2023; Mesfin et al., 2021; Rashwan et al., 2023). Among them, brown rice protein isolate (BRPI) as an important plant-derived protein resource, has gradually attracted widespread attention from food formulation experts and researchers due to its relatively balanced essential amino acid composition, low allergenicity, and cost-effectiveness (Chinma et al., 2023). However, despite these advantages, the interfacial adsorption capacity of native BRPI is relatively weak. This is mainly attributed to its relatively rigid internal structure, which stems primarily from the abundant *β*-sheets and *β*-turns within its molecules (Xu et al., 2023), as well as strong intermolecular hydrophobic interactions and disulfide bond cross-linking (Yin et al., 2026), leading to the formation of large aggregates in solution with a wide particle size distribution and low specific surface area (Sun et al., 2025). These structural characteristics hinder the anchoring of BRPI molecules at the oil-water interface, reducing its application potential as a natural plant-based emulsifying ingredient.

To overcome this bottleneck, scientists have devised multiple approaches in recent years to modifying the structural characteristics of proteins derived from rice. These methods aim to improve their emulsifying performance, including ultrasound-alkali-thermal synergistic treatment (Zhu et al., 2025), limited enzymatic hydrolysis combined with high hydrostatic pressure (Liu et al., 2022), deamidation using citric acid-based natural deep eutectic solvents (Shi et al., 2024), and individual treatments such as ultra-high pressure, microwave, phosphorylation, glycosylation, and hydrolysis with alkaline protease or papain (Sun et al., 2025). While these methods show some efficacy in improving emulsifying performance, they generally face challenges in industrial applicability, such as high energy consumption, potential chemical residue risks, or induction of off-flavors. In contrast, germination—a natural, endogenous, non-chemical, and low-energy biomodification process—aligns with the food industry's core demands for clean labels, green processing, and sustainable development (Liu et al., 2023). Notably, germination modification differs fundamentally from direct enzymatic hydrolysis: the latter relies on exogenous proteases (e.g., alkaline or flavor proteases) to perform controlled hydrolysis of substrate proteins (Gharehbeglou et al., 2024). Although this approach allows precise regulation of the degree of hydrolysis and effectively enhances solubility, emulsifying capacity, and foaming properties, it often leads to the release of bitter peptides and disruption of gel network integrity, thereby limiting its application in texture-sensitive food products such as meat analogues and gel desserts (Pang et al., 2024). Furthermore, this process requires the procurement of high-purity commercial enzymes, and strict control over reaction parameters (pH, temperature, and time), all of which significantly increase production costs and process complexity (Wu and Shin, 2026). Unlike direct enzymatic hydrolysis, germination is a critical physiological stage marking the reactivation of life activities in plant seeds, triggered by the perception and transduction of various environmental signals by internal tissue cells, and exerts a decisive influence on subsequent plant growth and development (Guzmán-Ortiz et al., 2018; He and Yang, 2013; Jhanji et al., 2024). This process typically involves three phases: (I) the water absorption phase, where grains or seeds are soaked in water, and moisture penetrates the seed interior through stomata and micropores, interacting with proteins and fibers for storage; (II) the phase of stable water absorption rate, during which the pericarp ruptures at the embryo region and the radicle begins to emerge; and (III) the radicle development phase, where water absorption rate rises again as radicle cells proliferate during growth (Do Nascimento et al., 2022; Xu et al., 2025). During germination, endogenous hydrolases (e.g., proteases, peptidases) are activated under the induction of gibberellins and other endogenous hormones. These hydrolases then diffuse into the endosperm, promoting the gradual degradation of storage proteins within it and triggering systematic remodeling of proteins from primary to higher-order structures (Do Nascimento et al., 2024), which significantly improves protein functional characteristics.

For instance, studies indicate that the germination process elevates both the concentration of free sulfhydryl groups and the surface hydrophobicity in sesame protein. These changes are recognized indicators of protein unfolding (Hu et al., 2023), potentially enabling more efficient interfacial adsorption and the development of interfacial films that are both stronger and more flexible, thereby enhancing the emulsification performance of food systems. Similarly, germinated black bean protein isolate exhibits reduced particle size, higher absolute zeta potential, and extended protein structure, which collectively enhance its emulsifying performance (Wang et al., 2024). Additionally, the emulsifying capacity and stability of emulsions formed with protein isolates from Indian teff are enhanced as germination progresses, likely due to partial protein hydrolysis that enhances solubility and reveals hydrophobic regions. Such modifications promote the protein's migration to the interface between oil and water, lower the interfacial tension, and facilitate the development of viscoelastic films at the interface, facilitating emulsion formation and stability (Niu et al., 2023, 2024, 2025; Sharma et al., 2026). However, existing studies primarily focus on bulk protein property changes, lacking systematic analysis of key interfacial processes of germinated BRPI, such as dynamic adsorption behavior, interfacial film rheological responses, and interfacial tension decay kinetics. These interfacial-scale dynamic properties are precisely the theoretical and technical foundation for elucidating the structure-function relationship of protein emulsification. Based on this, we hypothesize that the structural evolution pathway and extent of BRPI after germination may differ from other reported cereal proteins, leading to differential responses in oil-water interfacial adsorption kinetics, interfacial film mechanical behavior, and macroscopic emulsifying performance. Therefore, under strictly controlled temperature and humidity conditions, this study systematically investigates the effects of different germination durations (0–24 h) on the molecular structural characteristics of BRPI, oil-water interfacial adsorption behavior and kinetic parameters (interfacial tension/interfacial pressure, adsorption rate constant), interfacial film rheological properties (interfacial elastic modulus, viscous modulus, and viscoelastic characteristics), and emulsifying performance (emulsifying activity, emulsion stability). Furthermore, a three-dimensional correlation model of "protein structure–interfacial adsorption–emulsifying function" will be constructed. The findings are expected to provide theoretical basis and technical support for the precise functional application of BRPI in high-value food systems such as bakery products and plant-based milk beverages.

In summary, germination has been shown to enhance the emulsifying properties of sesame and black bean proteins by altering their structural and physicochemical characteristics. These findings suggest that germination-induced structural remodeling plays a common role in improving plant protein functionality. However, the extent and direction of these changes depend on the specific protein system and processing conditions. For rice-derived proteins, aggregation and limited aqueous solubility constitute significant barriers to their application as natural emulsifiers in the food industry. For instance, studies have reported that the solubility of native rice protein can be as low as 1.84% (Wang et al., 2026b). Consequently, it remains necessary to clarify how germination-induced changes in the dispersibility and conformational adaptability of BRPI are associated with adsorption at the oil–water interface and the mechanical properties of the resulting interfacial films. This study addresses this issue by integrating structural characterization, interfacial adsorption kinetics, dilatational rheology, and emulsifying performance assessments for untreated, soaked, and germinated BRPI. The significance of this work lies in elucidating the stage-dependent relationships among protein structure, interfacial adsorption, film mechanics, and emulsifying performance, moving beyond previous studies that only demonstrated changes in macroscopic emulsifying properties. Particular attention was paid to whether soaking and subsequent germination elicited distinct responses in adsorption kinetics and film elasticity, and how these responses correlated with the emulsifying activity index (EAI) and emulsifying stability index (ESI).

## 2 Materials and methods

### 2.1 Materials

The brown rice variety used in this study was Bingliangyou 518, provided by the Key Laboratory of Crop Physiology, Ecology and Genetic Breeding of the Ministry of Education and Jiangxi Province (Nanchang, China). Ultrapure water was produced using a system purchased from Merck (Darmstadt, Germany). Petroleum ether, sodium hydroxide, and hydrochloric acid were supplied by Xilong Scientific Co., Ltd. (Shantou, China). Trypsin (250 USP units/mg) and trichloroacetic acid were obtained from Macklin Biochemical Technology Co., Ltd. (Shanghai, China). The 0.05 mol/L Tris-HCl buffer (pH 8.0) and medium-chain triglycerides (MCT, 98% purity) were purchased from Yuanye Biotechnology Co., Ltd. (Shanghai, China), while potassium bromide was provided by Aladdin Biochemical Technology Co., Ltd. (Shanghai, China). Sodium dodecyl sulfate was obtained from Solarbio Science & Technology Co., Ltd. (Beijing, China). Florisil adsorbent and 1-anilino-8-naphthalenesulfonic acid (ANS) were purchased from Sigma-Aldrich (USA). Unless otherwise specified, all chemical reagents used in this study were of analytical grade.

### 2.2 Basic component analysis

The basic components of brown rice were determined following the method (Sharma et al., 2026). The moisture content was measured using AOAC method 930.15, and the protein content was determined via the Kjeldahl method (AOAC 984.13). The crude fiber content was estimated according to AOAC method 991.43. The crude fat content was analyzed using AACC method 30.25.01, while the ash content was determined by AACC method 44.19. The carbohydrate content was calculated by the difference method, i.e., subtracting the sum of moisture, protein, fat, fiber, and ash contents from 100%.

### 2.3 Germination treatment of brown rice

The germination experiment of brown rice was conducted with appropriate optimizations based on the method (Sharma et al., 2026). The specific procedures were as follows: brown rice was soaked in distilled water in a constant-temperature water bath at 28°C for 12 h to ensure full imbibition. After soaking, the rice was rinsed three times with ultrapure water, drained of surface moisture, and evenly spread on a sterile gauze-pre-wetted culture tray. It was then transferred to a constant-temperature and constant-humidity incubator (temperature: 30 ± 0.5°C, relative humidity: 85 ± 0.2%) for germination. The germination time gradients were set at 8 h, 16 h, and 24 h, with the corresponding treatment samples labeled as S3, S4, and S5, respectively. Meanwhile, S1 was set as the original brown rice without any treatment (Control sample), and S2 as the sample that only underwent 12 h soaking, to distinguish the effects of soaking from those of germination. After the treatment of all samples was completed, the samples were immediately pre-frozen at −80°C for 2 h, followed by vacuum freeze-drying. After freeze-drying, the obtained samples were sealed and stored at 4°C in the dark for subsequent physicochemical and functional property analyses.

### *2.4* *Germination rate of brown ric*e

The germination rate of BRPI samples was determined following a previously reported method (Sharma et al., 2026). All samples were prepared in triplicate, with each replicate containing 100 morphologically intact seeds free from obvious damage. A seed was considered germinated if the radicle had broken through the seed coat and was clearly visible. The germination rate (%) was calculated using the following formula (1):

(1) $$\begin{array}{c} Germination\;percentage\;\left(\%\right)=\frac{A}{B}\times 100 \end{array}$$

Where *A* is the number of germinated grains and *B* is the total number of grains tested.

### 2.5 Protease activity assay

The protease activity of all samples was determined with reference to (Li et al., 2010). Briefly, 2 g of frozen germinated seeds were taken, and 8 mL of 0.1 mol/L citrate-phosphate buffer (pH 6.0) containing 5 mmol/L *β*-mercaptoethanol was added in an ice bath for homogenization. The homogenate was centrifuged at 16,000×g for 30 min at 4°C, and the supernatant was collected for protease activity assay. Before the protease activity determination, a reaction system was prepared by dissolving 1 mL of 20 g/L casein solution and 1 mL of enzyme solution in 0.1 mol/L Tris-HCl buffer (pH 8.0). The reaction was carried out at 40°C for 10 min, and then terminated by incubating in a 90°C water bath for 5 min 2 mL of 0.4 mol/L trichloroacetic acid (TCA) was added to each test tube, and after cooling at 4°C for 30 min, centrifugation was performed at 12,000×g for 20 min. The protease activity was measured as the increase in absorbance of the TCA-soluble fraction at 275 nm using a UV-visible spectrophotometer (UNICO UV-2802, USA), and calibrated against the absorbance of tyrosine at 275 nm.

### 2.6 Extraction of BRPI

Prior to protein extraction, all samples were ground and sieved through a 60-mesh sieve to obtain germinated brown rice flour. A certain weight of the prepared germinated brown rice flour was weighed, and a 0.1 mol/L NaOH solution was added at a solid-to-liquid ratio of 1:6, followed by magnetic stirring at room temperature for 2 h. The mixture was then centrifuged at 8000×g for 15 min, and the protein supernatant was collected. A 0.1 mol/L HCl solution was slowly added to the protein supernatant until the pH dropped to 5.0, then centrifuged at 8000 g for 15 min to obtain protein precipitate. The acid-precipitated protein precipitate was adjusted to neutral pH using a 0.1 mol/L NaOH solution and washed three times with deionized water to obtain the germinated BRPI precipitate. The precipitate was then freeze-dried in a vacuum freeze dryer for 48 h, and the germinated BRPI samples were collected.

### 2.7 Determination of protein content

The protein content in the BRPI was determined strictly in accordance with the Kjeldahl method (*N*×5.95) (Li et al., 2022).

### 2.8 Determination of protein structure

#### 2.8.1 Sodium dodecyl sulfate-polyacrylamide gel electrophoresis (SDS-PAGE)

The molecular weight distribution of proteins was analyzed using SDS-PAGE. The experiment was performed with a DYY-6D vertical electrophoresis system (Beijing Liuyi Biotechnology Co., Ltd., China). Samples were mixed with 5× SDS loading buffer at a ratio of 4:1 (v/v) and denatured in a boiling water bath for 5 min. A 10 μL aliquot of the loading solution was loaded into the wells of a precast gel consisting of 4% stacking gel and 12% separating gel. Electrophoresis was conducted at a constant voltage of 120 V for 25 min, followed by a voltage increase to 160 V for another 25 min. After electrophoresis, the gel was rinsed three times with ultrapure water (5 min each time), then stained with Coomassie Brilliant Blue R-250 ultra-rapid staining solution at room temperature for 6 h. Subsequently, the gel was destained with a destaining solution (ethanol: acetic acid: water = 2.5:1:6.5, v/v/v) under shaking until the background was clear and the bands were distinct. Finally, destaining was terminated with ultrapure water, and the gel was imaged using a rapid chemical imaging system.

#### 2.8.2 Particle size and zeta potential

The particle size and zeta potential of the samples were measured via dynamic light scattering and electrophoretic light scattering, respectively, at 25°C (Zetasizer Nano ZS90 system, Malvern Instruments Ltd., UK) (Igartúa et al., 2024). The refractive indices of the samples and the dispersion medium were set to 1.54 and 1.33, respectively. The protein concentration in the solution was adjusted to 2 mg/mL, and each sample was measured in triplicate. The zeta potential values were calculated using the Henry equation (2), where *U*_*E*_ represents electrophoretic mobility; *ε* is the dielectric constant; *z* stands for the zeta potential; *ƒ(ka)* refers to Henry's function; *η* denotes viscosity.

(2) $$\begin{array}{c} U_{E}=\frac{2\varepsilon \;zƒ\left(ka\right)}{3\eta } \end{array}$$

#### 2.8.3 Solubility and turbidity

The solubility of BRPI was determined with slight modifications based on a previously reported method (Li et al., 2026), and calculated using formula (3). Briefly, the protein dispersion was centrifuged at 8000×g for 15 min, and the nitrogen content in the resulting supernatant was measured using a BCA protein kit. The solubility of the protein sample was calculated as the ratio of soluble nitrogen content in the supernatant to the total nitrogen content in the solution. Additionally, the turbidity of the protein solution (1 mg/mL) was characterized by measuring its absorbance at 600 nm using a spectrophotometer (UV-1280, Shimadzu, Japan), with pure water serving as the negative control.

(3) $$\begin{array}{c} \text{Solubility}\left(\%\right)=\frac{protein\;content\;in\;supernatant}{total\;protein\;content\;before\;centrifugation}\times 100\% \end{array}$$

#### 2.8.4 Fourier transform infrared spectroscopy

Following the method of previous research (Shu et al., 2025), freeze-dried BRPI samples were sieved through a 100-mesh sieve, then mixed with spectroscopically pure potassium bromide (KBr) at a ratio of 1:100 (w/w). The mixture was fully ground in an agate mortar until uniform and free of visible particles, then pressed into transparent pellets under a pressure of approximately 10 MPa using a tablet press. Infrared spectra were collected using a fourier transform infrared spectrometer (Thermo Nicolet, Madison, USA), with a scanning range of 4000–400 cm^−1^, 32 cumulative scans, and a resolution of 4 cm^−1^. Background scanning was performed synchronously prior to sample testing.

#### 2.8.5 Surface hydrophobicity

The surface hydrophobicity of all protein samples was determined using the fluorescent probe method (Li et al., 2026). First, a protein stock solution with a concentration of 2 mg/mL was prepared, and then serially diluted with phosphate-buffered saline (PBS, 10 mM, pH 7.0) to obtain a series of protein solutions with final concentrations of 0.025, 0.05, 0.1, 0.2, and 0.4 mg/mL, respectively. For each concentration, 4.0 mL of the solution was taken, and 20 μL of 8 mmol/L ANS solution was added. The mixture was vortexed for 30 s to homogenize and then incubated at room temperature in the dark for 30 min. The fluorescence intensity of the BRPI samples was measured using a fluorescence spectrophotometer (F2700, Hitachi High-Technologies Corporation, Tokyo, Japan). The detection parameters were set as follows: excitation wavelength at 390 nm, emission wavelength at 470 nm, and both excitation and emission slit widths at 5.0 nm. A standard curve was plotted with protein concentration as the abscissa and the corresponding fluorescence intensity as the ordinate. The slope obtained from linear regression analysis was defined as the surface hydrophobicity index of the samples.

#### 2.8.6 Wettability

The contact angle was measured using an optical video contact angle meter (OSA100, New Frontiers Scientific Instruments Co., Ltd., Ningbo, China) (Wu et al., 2024). Specifically, the freeze-dried BRPI powder was pressed into uniform, flat circular disc-shaped protein samples, which were then placed in a square glass dish containing oil. A 5 μL droplet was formed at the needle tip and deposited onto the sample surface, and images were captured 30 s thereafter.

#### 2.8.7 Conformational flexibility

The conformational flexibility of BRPI was determined following a previously reported method (Wang et al., 2026a). A 250 μL enzyme solution (1 mg/mL, dissolved in 0.05 mol/L Tris-HCl buffer at pH 8.0) was mixed with 4 mL of a 1 mg/mL protein solution diluted with deionized water (trypsin-to-protein mass ratio of 1:16, w/w); the mixture was then immediately incubated in a 38°C water bath for 5 min. After the reaction, 4 mL of 5.0% (w/v) trichloroacetic acid (TCA) was added to terminate the enzyme activity. The reaction mixture was centrifuged at 8000×g for 15 min, and the supernatant was collected. A spectrophotometer (UV-5200PC, Shanghai Yuanxi Instrument Co., Ltd.) was used to measure the absorbance of the supernatant at 280 nm, and the measured absorbance value (*A*_*0*_) was finally used as a quantitative indicator of molecular flexibility.

#### 2.8.8 Scanning electron microscope (SEM)

For all treated samples subjected to freeze-drying, they were first affixed to aluminum sample stubs using conductive double-sided tape, followed by gold sputter coating under a high-vacuum environment. Subsequently, a field-emission scanning electron microscope (Carl Zeiss EVO18, Zeiss Group, Germany) was used to observe and analyze their surface micromorphology. All images were captured under the secondary electron imaging mode at a magnification of 200×.

### 2.9 Determination of adsorption behaviors at the oil–water interface

#### 2.9.1 Interfacial tension and interfacial pressure

The interfacial tension was measured via the pendant drop method using an interface analyzer (OSA100, New Frontiers Scientific Instruments Co., Ltd., Ningbo, China) (Wang et al., 2026a). For the oil–water interface, the reaction cell was pre-filled with molecularly distilled medium-chain triglycerides (MCT) as the oil phase. Subsequently, a 10 μL aqueous phase droplet was precisely injected into the oil phase using a microsyringe to form a stable pendant drop. Immediately after the droplet formed, the high-speed imaging system was activated to continuously record the droplet profile for 10,000 s. The dynamic interfacial tension values at each time point were obtained by fitting the droplet shape parameters using the *Young–Laplace* equation. The interfacial pressure (*π*) was calculated using equation (4):

(4) $$\begin{array}{c} \pi =\gamma_{0}-\gamma \end{array}$$

Here, *γ*_*0*_ = 22.5 mN/m corresponds to the equilibrium interfacial tension between pure water and MCT at 25°C; γ denotes the dynamic interfacial tension measured at the oil–water interface for a 10 mg/mL BRPI solution.

#### 2.9.2 Interfacial adsorption kinetics

Typically, the initial stage of the adsorption process is primarily governed by the diffusion rate of the protein, which is characterized by the interfacial diffusion rate (*K*_*d*_, mN·m^−1^·s^−0.5^) and derived using equation (5). In this equation, *C*_*0*_ denotes the protein concentration in the solution (g/mL); *K* is the Boltzmann constant; *T* represents the absolute temperature; *D* is the diffusion coefficient of the protein; and *t* stands for the adsorption time (s).

To quantitatively analyze the two-stage kinetic behavior of protein molecules at the oil-water interface—penetration (*K*_*p*_) and conformational rearrangement (*K*_*r*_)—equation (6) is applied. Here, *π*_*10000*_, *π*_*0*_, and *π*_*t*_ correspond to the interfacial pressure at 10,000 s, initial time (0 s), and any arbitrary time (s), respectively; *K*_*i*_ is the first-order rate constant. The plot generated from equation (6) exhibits two distinctly separated linear segments: the slope of the initial linear segment corresponds to the penetration rate constant of the protein at the interface (*K*_*p*_, s^−1^), while the slope of the subsequent linear segment reflects the kinetic rate of molecular conformational rearrangement after adsorption is completed (*K*_*r*_, s^−1^).

(5) $$\begin{array}{c} \pi =2\cdot C_{0}\cdot K\cdot T\cdot {\left(\frac{Dt}{3.14}\right)}^{\frac{1}{2}} \end{array}$$

(6) $$\begin{array}{c} \ln\left[\frac{\left(\pi_{10000}‐\pi_{t}\right)}{\left(\pi_{10000}‐\pi_{0}\right)}\right]=-k_{i}t \end{array}$$

#### 2.9.3 Interfacial dilatational rheology

Following the dynamic interfacial tension measurement, amplitude sweep and frequency sweep were performed separately (Zhan et al., 2019). The amplitude sweep was conducted at a constant frequency of 0.1 Hz, with the amplitude gradually increasing from 1.5% to 30%. The frequency sweep was carried out at a constant amplitude of 10%, over a frequency range of 0.005 to 0.1 Hz. Furthermore, to more realistically simulate the oil-water interfacial behavior of protein molecules in complex food systems and deeply explore the structure-activity relationship between interfacial dilatational rheological properties and macroscopic emulsion stability, this study further plotted Lissajous figures based on nonlinear interfacial dilatational rheology data (Zhao et al., 2026). The Lissajous curves were constructed from the output of the interface analyzer (OSA100, New Frontiers Scientific Instruments Co., Ltd., Ningbo, China), reflecting the relationship between interfacial pressure (*π* = *γ*_*0*_ *- γ*) and surface deformation (*δA/A*_*0*_*, where δA* = *A – A*_*0*_) during the amplitude sweep. In addition, for linear interfacial dilatational rheology, the area of the 10 μL droplet was sinusoidally oscillated at a constant frequency of 0.1 Hz and a constant amplitude of 10% (Wang et al., 2026a), with the experiment duration set to 10,000 s and the experimental temperature maintained at 25 ± 0.5°C.

### 2.10 Characterization of emulsion properties

#### 2.10.1 Determination of emulsifying activity and emulsion stability

The emulsifying properties of BRPI were determined according to a previously reported method (Wang et al., 2025). Specifically, 100 μL of the emulsion sample was thoroughly mixed with 5 mL of 0.1% (w/v) sodium dodecyl sulfate (SDS) solution. Using an equal volume of SDS solution as the control, the initial absorbance (*A*_*0*_) and the absorbance after 10 min of standing (*A*_*10*_) of the resulting solution were measured at a wavelength of 500 nm using a UV spectrophotometer (UV-5200PC, Shanghai Yuanxi Instrument Co., Ltd., China), followed by calculation via (7), (8). Here, *N* represents the dilution factor (51), *C* denotes the protein concentration (g/mL), and *θ* is the oil phase fraction (0.2).

(7) $$\begin{array}{c} \text{EAI}\left(\frac{m^{2}}{g}\right)=\frac{2\times 2.303\times A_{0}\times N}{C\times \theta \times 10000} \end{array}$$

(8) $$\begin{array}{c} \text{ES}I\left(\min\right)=\frac{A_{0}}{A_{0}‐A_{10}}\times 10 \end{array}$$

#### 2.10.2 Determination of emulsion microstructure

The microstructure of the prepared emulsion was characterized using an optical microscope (Opto-Electronic Technology Co., Ltd., Chongqing, China). A 100 μL sample was uniformly dropped onto a clean glass slide, and a coverslip was placed on top to avoid bubble introduction and sample evaporation. The slide was then placed under the optical microscope, and bright-field observation and image acquisition were performed using a 10× objective lens.

### 2.11 Statistical analysis

All experiments were conducted with three replicates, and the data are presented as the mean ± standard deviation. Statistical analysis was performed using SPSS 26.0 software (IBM Corporation, Armonk, NY, USA), employing one-way analysis of variance (ANOVA) followed by Duncan's multiple range test, with the confidence level set at 95% and a *p*-value < 0.05 considered statistically significant. In addition, Origin 2025 software was used for graphing and for calculating the Pearson correlation coefficients among the variables.

## 3 Results and discussion

### 3.1 Basic components of brown rice

To offer a scientific foundation for the subsequent structural characterization of BRPI, the basic components of brown rice were determined. The results showed that brown rice contained 74.52% carbohydrates, 9.24% protein, 2.68% fat, 0.98% dietary fiber, 1.24% ash, and 11.34% moisture content. These findings align with earlier studies regarding the basic components of brown rice, confirming its relatively rich nutritional effects (Saleh et al., 2019).

### 3.2 Germination rate

The germination rate is a vital parameter for assessing the vigor of brown rice seeds, and its level potentially affects the processing properties, nutritional value, and final product quality of brown rice (Kim et al., 2022). Therefore, the germination rates of samples subjected to different germination treatments were characterized. Fig. 1A shows the appearance and morphology of the samples after germination under different times. It is evident that since the S1 sample was not soaked and germinated under specific conditions, the synthesis of the endogenous hormone abscisic acid was much higher than that of gibberellin, and the seeds were generally in a dormant state (Yan and Chen, 2020). Further observation revealed that the S2, S3, S4, and S5 samples had relatively smooth and plump appearances, and gradually showed visible buds, with typical bud distribution observed in the S5 sample. This pattern was also verified in Fig. 1D, where the germination rates from low to high were 0% (S1) < 2.33% (S2) < 10.67% (S3) < 48% (S4) < 78.33% (S5). This observation aligns with earlier findings regarding the germination patterns of *Eragrostis tef* seeds, they found that as the germination time gradually increased, the germination rate of seeds significantly increased, reaching an almost full germination state (99%) when the germination period reached 24 h (Sharma et al., 2026). However, in this study, the germination rate of brown rice seeds was lower (78%) when the germination period reached 24 h, which may be attributed to differences in variety and genotype.

**Fig. 1:**
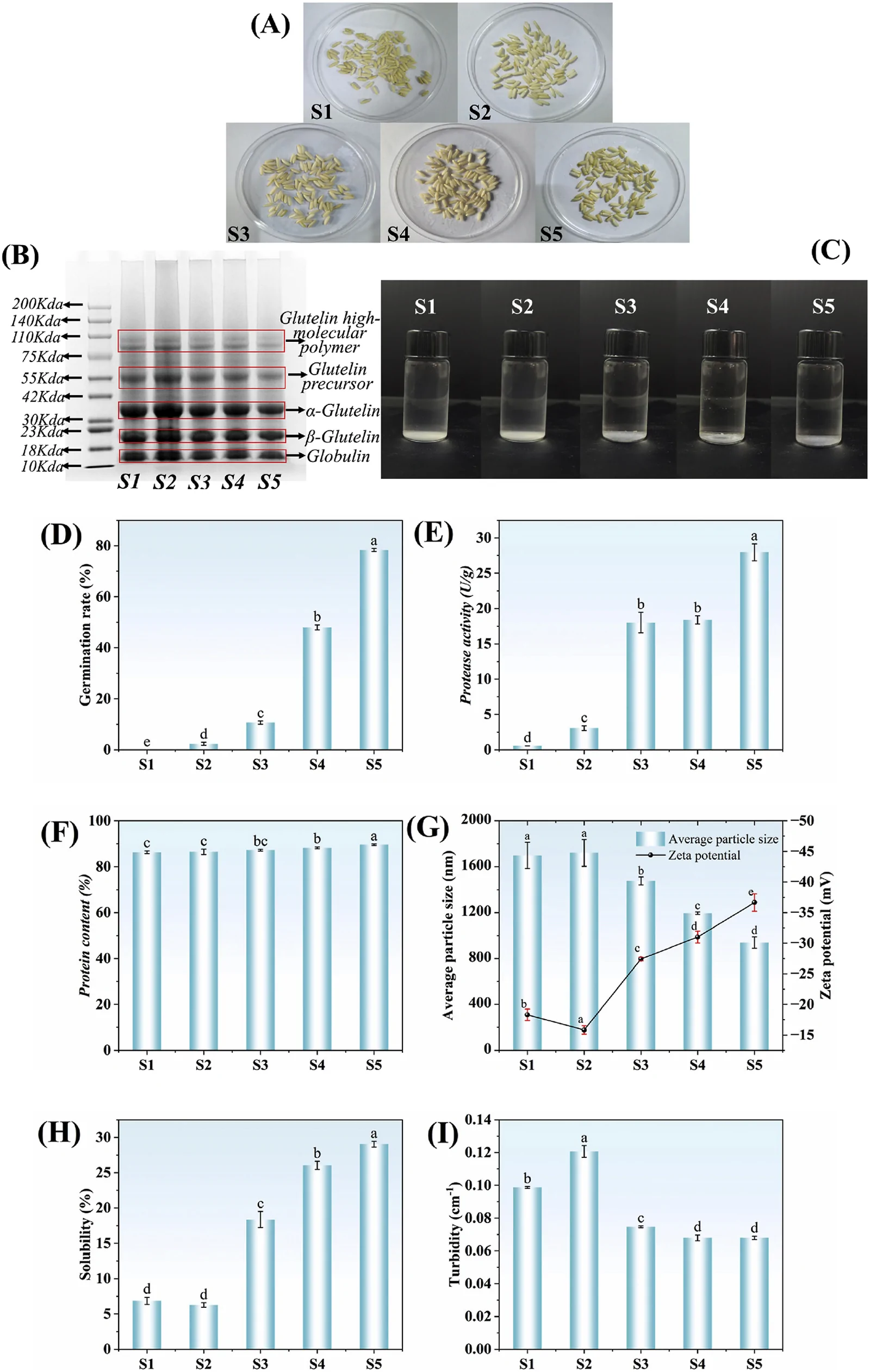
Effects of different germination times on the appearance and morphology of brown rice (A), as well as the molecular weight distribution (B), solution state (C), germination rate (D), protease activity (E) protein content (F), particle size and zeta potential (G), and solubility (H) and turbidity (I) of BRPI.

### 3.3 Protease activity

Typically, during plant seed germination, stored nutrients—particularly storage proteins—undergo systematic hydrolysis, a process primarily catalyzed by coordinated protease action. This serves to supply essential nitrogen sources, carbon skeletons, and energy for early seedling growth (Farooq et al., 2022). Accordingly, we further conducted dynamic tracking of protease activity in brown rice during germination. As shown in Fig. 1E, there were significant differences in protease activity intensity among the samples. Specifically, sample S1 exhibited relatively low protease activity (0.57 U/g) compared to other samples. This phenomenon is closely linked to its lack of pre-soaking treatment: saturated water absorption is a prerequisite for activating endogenous hormones such as gibberellins, which in turn induce protease expression and activation. Without this critical step, the enzyme activity of S1 was significantly suppressed. In contrast, the fully soaked Sample S2 showed a marked increase in protease activity (3.07 U/g), indicating that relief from water stress effectively triggered the endogenous hormone signaling pathway, leading to a sharp rise in protease activity. Further analysis revealed that protease activity continued to increase in germinated samples (S3–S5), peaking at 27.97 U/g in sample S5 after 24 h of germination. Similar trends have been reported in related studies on protease activity in germinated brown rice, where it is proposed that this enhancement may stem from gibberellins produced in the embryos of germinating seeds, which promote protease formation (Li et al., 2010). This likely explains the increased protease activity during brown rice germination in the present study, and such changes may alter the protein structure of brown rice.

### 3.4 Content of BRPI

The changes in protein content of BRPI subjected to different germination times are shown in Fig. 1F. As indicated in Fig. 1F, there were significant differences in protein content among the treatment samples (*p* < 0.05). Specifically, the protein content of the unsoaked and ungerminated S1 sample was relatively low (86.30%). Interestingly, with the gradual increase in germination time (0-24h), the protein content of the BRPI showed a continuous upward trend, reaching a peak in the S5 sample (89.58%). This phenomenon can be attributed to the activation of endogenous proteolytic enzymes during germination; such enzymatic reactions are primarily mediated by protein bodies enriched in the seed coat and embryonic axis tissues, which in turn promote the development of new tissues and significantly increase the content of proteins (Medhe et al., 2023; Mesfin et al., 2021; Sofi et al., 2020). A similar trend was observed in sesame protein isolate, where the sesame protein content slightly increased after 24 h of soaking and germination (Hu et al., 2023). In conclusion, these results suggest that short-term germination treatment in this study can increase the protein content of BRPI, which may provide a potential approach to improve protein recovery.

### 3.5 SDS-PAGE

To elucidate the impact of varying germination durations on the molecular weight derived from BRPI, the molecular characteristics were further characterized by SDS-PAGE. As shown in Fig. 1B, all analyzed samples displayed five distinct bands in their respective lanes during electrophoresis, located around 10–18 kDa, 18–23 kDa, 30–42 kDa, 55 kDa, and 75–110 kDa, corresponding sequentially to globulin, *β*-glutelin, *α*-glutelin, glutelin precursor, and glutelin high-molecular polymer of BRPI (Ghanghas et al., 2020; Song et al., 2024). The main bands were distributed between 18 and 42 kDa. Further analysis revealed notable variations in the intensity of electrophoretic bands across the various specimens. Specifically, compared with the S1 sample, the S2 sample exhibited higher intense band signals. This phenomenon may suggest that soaking treatment initially promotes the aggregation of BRPI. Interestingly, the intensity of electrophoretic bands further decreased with the increase in germination time (8–24 h), especially the bands around 55 kDa and 75–110 kDa, which weakened more severely and tended to disappear in the S5 sample. This indicates that the storage proteins in brown rice may undergo hydrolysis during the germination process due to the enhanced activity of proteases, which is consistent with the changes in protease activity (Fig. 1E). This trend is consistent with the results of sesame protein and *Eragrostis tef* protein treated under similar conditions (Hu et al., 2023; Sharma et al., 2026). In conclusion, germination treatment significantly altered the primary structure of BRPI, mainly manifesting as the hydrolytic degradation of natural storage proteins. This structural change may further affect its oil-water interface adsorption properties.

### 3.6 Particle size and zeta potential

Generally, particle size can characterize the aggregation state and degree of protein molecules, while zeta potential indicates protein stability in a dispersion system (Li et al., 2026). Fig. 1G illustrates the distribution of mean particle size and zeta potential of BRPI across various germination periods. As shown in the figure, the average particle size for sample S2 (1720.67 nm) is slightly larger than that of sample S1 (1698.12 nm), indicating that short-term soaking may induce initial aggregation of proteins. However, as the duration of germination was progressively increased from 8 to 24 h, the mean particle size of BRPI gradually decreased, reaching the minimum value (938.85 nm) in the S5 sample. This suggests that germination treatment leads to protein depolymerization. This structural change may be due to the activation of endogenous proteases during germination (as shown in Fig. 1E); these enzymes can partially hydrolyze proteins, making the structure of originally large protein aggregates more open and looser, and promoting the exposure of charged samples originally buried within the protein. This imparts a higher electrostatic repulsion on the protein surface, which overcomes the intermolecular attraction and aggregation, leading to a significant reduction in the mean particle size of BRPI (Hu et al., 2023). This mechanism can in turn reasonably explain the relatively large particle size of the S2 sample. The above inference is further supported by the zeta potential (Fig. 1G): the absolute value of zeta potential in the S2 sample (−15.82 mV) was significantly lower than that in the S1 sample (−18.30 mV), while with the progress of germination (8–24 h), its absolute value continued to increase, reaching the highest value (−36.65 mV) in the S5 sample. Previous studies have shown that the higher the absolute value of zeta potential, the stronger the electrostatic repulsion between particles, which is more conducive to overcoming van der Waals attraction, thereby effectively inhibiting protein aggregation, flocculation, or sedimentation (Wang et al., 2023). This further explains why the mean particle size of the S5 sample is much smaller than that of the S2 sample. In conclusion, germination treatment can not only alleviate the aggregation degree of BRPI by enhancing electrostatic repulsion, but also help improve its colloidal stability. Such improvements are crucial for supporting both the formation and the long-term stability of emulsions (Saricaoglu et al., 2018).

### 3.7 Solubility and turbidity

Solubility is not only a key physicochemical parameter for evaluating the aggregation tendency and denaturation degree of proteins, but also has a significant impact on their emulsifying properties (Wang et al., 2025). Therefore, the solubility of BRPI treated with different germination times was characterized. As shown in Fig. 1H, different germination times have a significant regulatory effect on the solubility of BRPI. Specifically, compared with the S1 sample (6.86%), the solubility of the S2 sample slightly decreased (6.27%), which may be due to the weakening of electrostatic repulsion caused by the fine-tuning of protein conformation in the early soaking stage, inducing mild intermolecular aggregation. This is consistent with the results of its mean particle size and zeta potential (Fig. 1G). Further studies found that the prolonging of germination time (8–24 h) can improve the solubility of BRPI, reaching a peak in the S5 sample (29.03%). This maximum improvement in solubility is likely due to the proteases in brown rice being activated under this condition (as shown in Fig. 1E), which promotes the degradation of BRPI. This change leads to the exposure of surface charges, thereby enhancing intermolecular electrostatic repulsion, resulting in the depolymerization of originally large-sized aggregated BRPI into smaller particle sizes. Such smaller particle sizes usually have a larger specific surface area, which enhances contact with water molecules (Li et al., 2023). Therefore, the solubility of the S5 sample was significantly improved. This explanation is also confirmed by the molecular weight (Fig. 1B) and particle size and zeta potential (Fig. 1G).

Furthermore, turbidity was characterized as another indicator of the aggregation state of protein molecules. Fig. 1C displays the photographs of sample solutions at different germination times. Visually, there are discernible differences among the samples: compared to S1, the S2 solution appears relatively more turbid with a greater amount of precipitate. However, as germination time prolonged, the turbidity of the sample solutions gradually decreased, and precipitation was also alleviated, suggesting that germination treatment enhances the solubility of BRPI—a change in solubility typically accompanied by a reduction in turbidity (Li et al., 2026). Nevertheless, such visual observations may be subject to observer bias; therefore, they should serve only as preliminary references, necessitating further quantitative analysis of turbidity across different germination times. As shown in Fig. 1I, the turbidity trend is inverse to that of solubility, initially rising and subsequently declining. Specifically, the turbidity of sample S2 (0.121) was higher than that of sample S1 (0.099), further indicating a relatively higher degree of aggregation in its solution. This is because when proteins aggregate in solution, larger and more uneven particles are formed, and these particles have significantly enhanced light scattering ability, thus leading to an increase in turbidity (Liu et al., 2017). Subsequently, with the extension of germination time (8-24 h), the turbidity of BRPI gradually decreased, which is a direct manifestation of protein depolymerization and improved colloidal stability (Feng et al., 2024). Further studies found that after more than 16 h of germination (S4 and S5 samples), the turbidity of BRPI no longer decreased significantly, indicating that germination treatment led to significant depolymerization of BRPI with better colloidal stability. This can be explained by the uncovering of surface charges following germinate, which facilitates the creation of a homogeneous protein dispersion. This observation aligns with the findings from zeta potential (Fig. 1G). In conclusion, germination treatment can not only enhance the solubility of BRPI, but also reduce the turbidity of its solution, which is vital for achieving effective adsorption at the interface between oil and water phases.

### 3.8 Secondary structure and conformational flexibility

When protein molecules adsorb at the oil-water interface, conformational changes typically occur to reduce interfacial free energy and form a stable interfacial film, among which changes in secondary structure are a key link (Zhan et al., 2022). Fig. 2A shows the distribution of secondary structure components of BRPI in the amide I band region (1700–1600 cm^−1^) after deconvolution and multi-peak fitting under different germination times. The corresponding amide I band spectra are shown in Supplementary Fig. S1. Within the amide I region, absorption peaks in the range of 1600–1640 cm^−1^ correspond to *β*-sheet structures; 1640–1650 cm^−1^ to random coil structures; 1650–1658 cm^−1^ to *α*-helix structures; and 1660–1700 cm^−1^ to *β*-turn structures (Wang et al., 2016). It is evident that all samples exhibit a secondary structure distribution dominated by *β*-sheets and *β*-turns, a similar finding reported in germinated sesame protein (Hu et al., 2023). Further analysis demonstrated that relative to the S1 sample, the *β*-sheet content in the S2 sample rose substantially to 45.82%, while the *β*-turn content dropped correspondingly to 22.15%. This suggests that the soaking treatment promoted a higher degree of structural order and rigidity in BRPI. However, as the germination period was prolonged from 8 to 24 h, the *β*-sheet content showed a continuous downward trend, while the *β*-turn content increased synchronously, with the lowest *β*-sheet content (37.56%) and highest *β*-turn content (33.60%) observed in the S5 sample. This suggests that germination treatment under this condition reduces the ordered structure of the protein, weakens its rigidity, and helps increase the conformational freedom of the main chain, making the protein molecules more flexible (De Brevern, 2022; Lian et al., 2022).

**Fig. 2:**
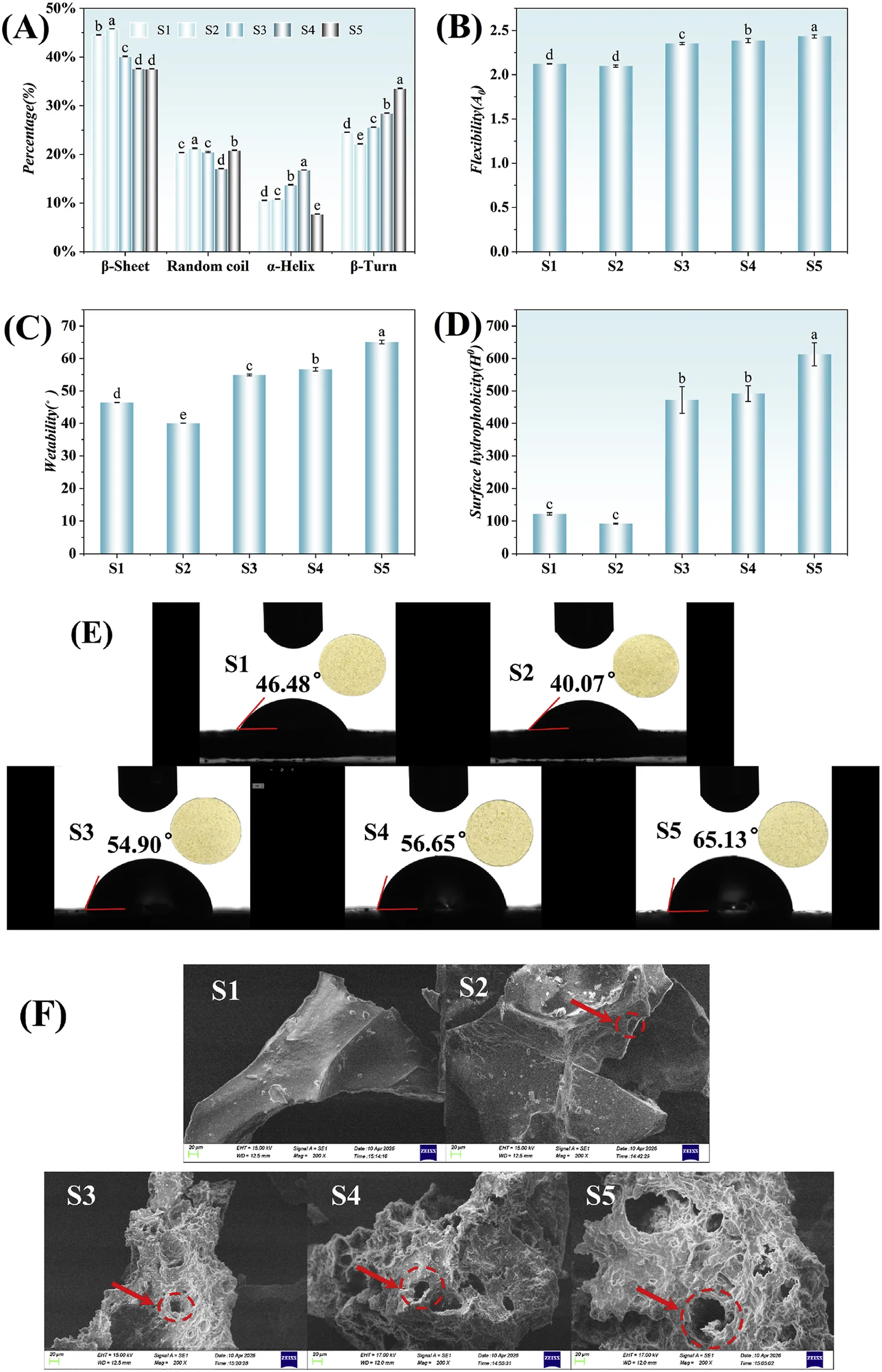
Effects of different germination times on the secondary structure (A), conformational flexibility (B), wettability (C), surface hydrophobicity (D), interfacial contact angle (E), and appearance morphology (F) of BRPI. The red markers in the appearance morphology images indicate the changes in the typical pore structure formed by BRPI during germination.

Following this, the conformational flexibility of BRPI samples was characterized. Compared with more rigid and compact proteins, proteins with higher conformational flexibility usually have faster interfacial adsorption kinetics, which is attributed to their easier conformational unfolding and molecular reorganization at the oil-water interface (Cai et al., 2023). Fig. 2B shows the changes in conformational flexibility of BRPI after different germination time treatments. Compared with the S1 sample (2.12), the conformational flexibility of the S2 sample slightly decreased (2.10), consistent with its rigid structural characteristics of high *β*-sheet and low *β*-turn content. Interestingly, with the extension of germination time (8–24 h), the conformational flexibility of BRPI was significantly improved, reaching a peak (2.43) in the S5 sample after 24 h of germination, indicating that germination treatment can significantly enhance the conformational flexibility of BRPI. This structural improvement can be explained by the partial hydrolysis of BRPI by endogenous proteases during the germination process (as shown in Fig. 1E), which reverses its original highly ordered structural characteristics of high *β*-sheet and low *β*-turn distribution, forming a distribution pattern of low *β*-sheet and high *β*-turn content. This is confirmed by the results of SDS-PAGE (Fig. 1B) and secondary structure (Fig. 2A). Furthermore, enhanced conformational flexibility might be strongly associated with alterations in the protein's surface hydrophobicity (Yan et al., 2021).

In conclusion, germination treatment can significantly regulate the secondary structure composition of BRPI, this regulation is primarily reflected in a reduction of *β*-sheet structures alongside a concurrent rise in *β*-turn components. This structural rearrangement helps enhance the conformational flexibility of protein molecules and provides the necessary structural basis for their rapid adsorption, unfolding, and stable film formation at the oil-water interface.

### 3.9 Wettability and surface hydrophobicity

The three-phase contact angle (*θ*) serves as a macroscopic measure that reflects the interfacial wettability (i.e., amphiphilic balance) of protein solid particles, which directly determines their adsorption affinity and orientation at the interface between oil and water phases (Wu et al., 2024). The impact of varying germination durations on the wettability of BRPI is illustrated in Fig. 2C. It is evident that different germination times have significant effects on its wettability. Compared with the S1 sample (*θ* = 46.48°), the wettability of the S2 sample decreased significantly (*θ* = 40.07°), indicating that soaking treatment impairs the hydrophobicity of BRPI, which may be detrimental to its adsorption at the interface between oil and water. Further studies revealed that with the gradual extension of germination time (8–24 h), the wettability of BRPI increased significantly, reaching the maximum value (*θ* = 65.13°) in the S5 sample. This trend aligns with the interfacial contact angle (Fig. 2E), implying that germination treatment can promote the exposure of hydrophobic samples originally hidden inside the core of BRPI to its surface, thereby improving its hydrophobicity. This phenomenon likely results from the substantial activation of internal proteolytic enzymes within the brown rice as it germinates under these specific conditions (as shown in Fig. 1E). Such enzymatic activity leads to the partial breakdown of proteins (Do Nascimento et al., 2024), which in turn loosen the compact structure of proteins. Consequently, hydrophobic amino acid residues that were initially buried within the molecular interior become more exposed (Hu et al., 2023; Li et al., 2023). Such appropriate interfacial wettability can not only effectively promote the adsorption and anchoring of particles at the oil-water boundary but also induce the development of a dense and stable three-dimensional steric hindrance layer at the interface, thereby significantly suppressing droplet coalescence (Tzoumaki et al., 2011).

However, wettability can only characterize the hydrophilic-hydrophobic properties of protein solid particles at the macroscopic level, and surface hydrophobicity at the molecular level must be determined by the ANS method. Therefore, the surface hydrophobicity of BRPI in solution was further determined. As shown in Fig. 2D, the surface hydrophobicity of BRPI was influenced by varying germination durations. A trend analogous to that of wettability was observed. Specifically, the surface hydrophobicity of the S2 sample exhibited a slight reduction to 92.47, compared to the value of 122.18 for the S1 sample. This decrease could be explained by the soaking treatment, which reduced the electrostatic repulsion of BRPI in solution (Fig. 1G). Consequently, the protein became more susceptible to aggregation, leading to the burial of certain hydrophobic regions within the molecular structure. However, with the gradual extension of germination time (8–24 h), its surface hydrophobicity gradually improved, showing relatively high surface hydrophobicity (612.79) in the S5 sample. This trend corresponds to the results of wettability (Fig. 2C). Previous studies have also shown that germination can significantly increase the surface hydrophobicity of sesame protein, which was attributed to the fact that enzymatic hydrolysis during seed germination can induce partial unfolding of protein conformation, resulting in the exposure of hydrophobic regions originally embedded in the tertiary structure of proteins to the solvent environment, thereby enhancing surface hydrophobicity (Hu et al., 2023). This may also be an important reason for the improvement of surface hydrophobicity in this study.

In conclusion, these results further confirm that the internal architecture of BRPI is substantially altered after germination treatment, and these changes may significantly affect its adsorption behavior at the oil-water interface, thereby regulating the formation and stability of subsequent emulsions.

### 3.10 Scanning electron microscopy (SEM)

SEM, a high-resolution surface imaging technique, is mainly used to characterize the macroscopic morphology, surface features, and aggregation state of proteins. Such structural information directly determines key functional properties of proteins, such as solubility and emulsifying properties (Li et al., 2026). Fig. 2F shows the SEM images of BRPI at a magnification of 200× under different germination times. The results indicate that germination significantly affects its surface morphology. Specifically, the surface of the S1 sample is relatively smooth and dense, with no obvious pores. However, as the germination time gradually increases (0–24 h), the appearance of the BRPI samples gradually shows obvious loose and porous structures, suggesting significant morphological changes in the protein matrix. Further studies found that when the germination time reaches 24 h, the S5 sample exhibits larger pores in its appearance. This dramatic morphological change indicates that the activity of endogenous proteases may be significantly enhanced during this germination stage, thereby exerting a substantial hydrolytic effect on the protein matrix, which further verifies the results of its primary structure (Fig. 1B) and protease activity (Fig. 1E). The resulting partial unfolding and degradation of proteins can effectively improve their solubility (Fig. 1H) and interfacial activity (Fig. 3A/B). A similar finding was reported in the effect of germination on the microstructure of barley protein (Román-Gutiérrez et al., 2026), where the protein network became loose but no obvious pores were observed. This difference may be attributed to variety specificity, depending on the genetic background of the grain and germination conditions (Kaur and Gill, 2020).

**Fig. 3:**
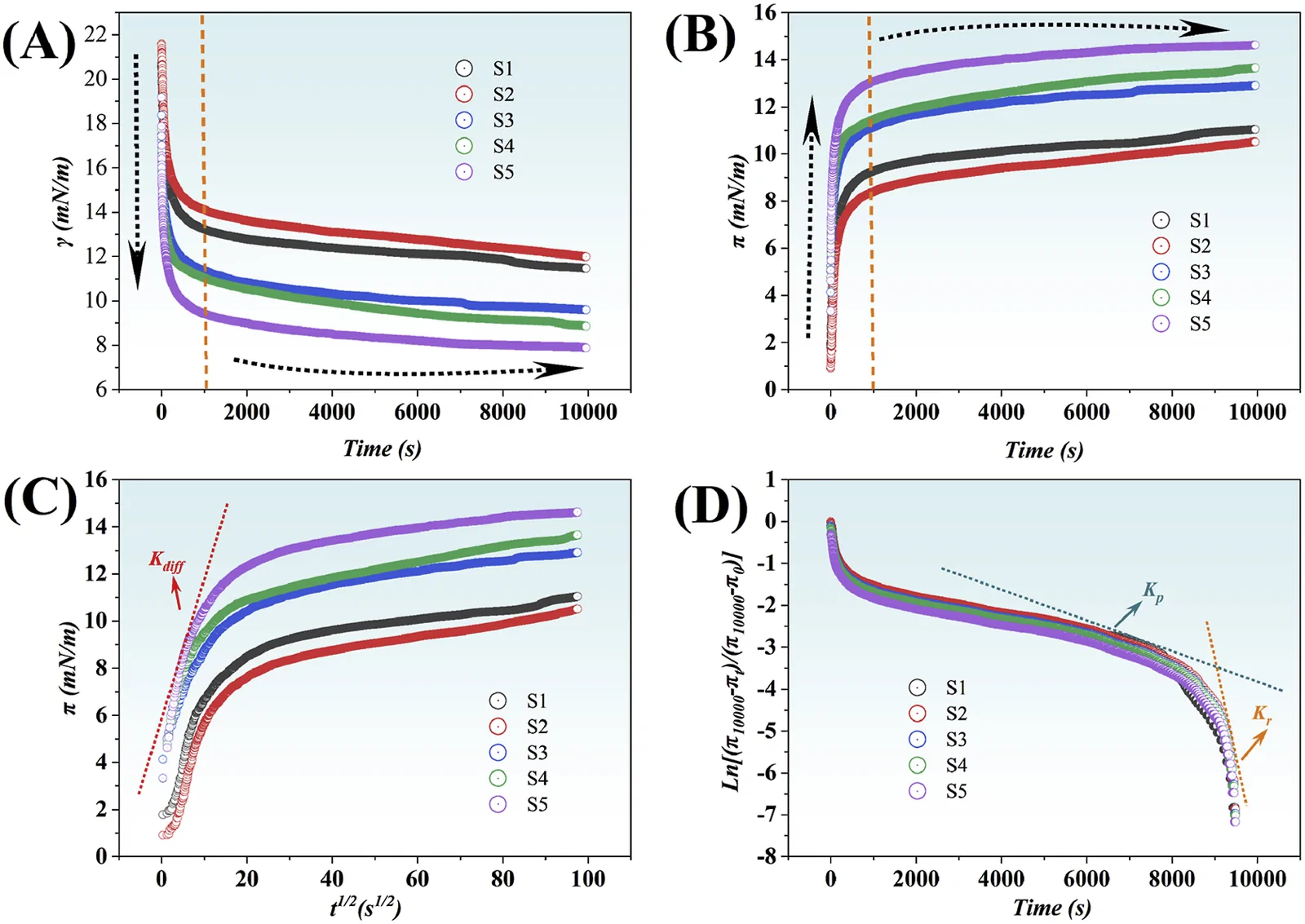
Effects of different germination times on the interfacial tension (A), interfacial pressure (B), and interfacial adsorption kinetics (C, D) of BRPI at the oil-water interface.

### 3.11 Interfacial tension

As a natural surface-active macromolecule with amphiphilic properties, the adsorption kinetics of BRPI at the oil-water interface significantly affects the dynamic evolution of interfacial tension and interfacial pressure; this influence mainly stems from the conformational rearrangement of protein molecules to adapt to the interfacial microenvironment (Zhang et al., 2022). Fig. 3A/B show the effects of different germination times on the interfacial tension (*γ*) and interfacial pressure (*π*) of BRPI at the oil-water interface. Overall, the change trends of interfacial tension/interfacial pressure of all samples are generally similar. Initially, with the progressive extension of adsorption time, a sharp decline in interfacial tension is observed alongside a swift increase in interfacial pressure. Subsequently, as the adsorption process continues over time, both the interfacial tension and interfacial pressure gradually approach and eventually attain a state of equilibrium. This trend is similar to the change trend of interfacial tension of temperature-regulated modified rice glutelin after pH shifting, as well as high-speed shearing studied by our team previously (Wang et al., 2025, 2026a), implying that in the initial stage of interfacial adsorption, BRPI first diffuses from the bulk solution to the oil-water interface. Subsequently, as the adsorption time further increases, more BRPI molecules adsorb at the oil-water interface, which not only leads to the saturation of adsorption sites at the interface but also enhances the electrostatic repulsion and steric hindrance between the protein molecules adsorbed at the interface (Wang et al., 2024; Wu et al., 2024; Zhu et al., 2024). Therefore, the interfacial tension of BRPI molecules at the oil-water interface shows a dynamic change process of first dropping sharply then slowly decreasing, while the interfacial pressure first rises sharply then slowly increases.

Interestingly, samples with different germination times show different change trends in interfacial tension and interfacial pressure at the oil-water interface. Specifically, compared with the S1 sample, the S2 sample has higher interfacial tension and lower interfacial pressure, this observation can be linked to the soaking treatment, which results in BRPI particles with a larger size and diminished solubility (Fig. 1G/H), these altered properties hinder the effective anchoring of BRPI at the oil-water interface. However, as germination time is progressively extended from 8 to 24 h, the ability of BRPI to reduce interfacial tension and increase interfacial pressure gradually improves, indicating that germination treatment is an effective method to enhance the interfacial activity of BRPI, allowing it to adsorb efficiently at the oil-water interface. Further studies revealed that the S5 sample has the strongest ability to reduce interfacial tension and enhance interfacial pressure at the oil-water interface, decreasing from 19.17 mN/m to 7.88 mN/m and increasing from 3.33 mN/m to 14.62 mN/m, respectively. This indicates that germination treatment under this condition can further enhance the interfacial adsorption capacity of BRPI. The above improvement in interfacial properties may be linked to the structural and physicochemical modifications of the protein brought about by germination. Firstly, the significant reduction in particle size and obvious improvement in solubility (Fig. 1G/H) are conducive to more soluble BRPI molecules diffusing and adsorbing to the oil-water interface more quickly and fully (Hu et al., 2023; Huang et al., 2024). Secondly, the enhanced surface wettability of the protein and the increased exposure of hydrophobic samples (Fig. 2C/D) further strengthen its directional anchoring and stable adsorption at the oil-water interface, thus providing a dual driving force for the improvement of interfacial activity (Tzoumaki et al., 2011; Wu et al., 2024).

In conclusion, germination treatment can significantly enhance the interfacial activity and adsorption capacity of BRPI at the oil-water interface. This effect is directly reflected in the significant reduction of interfacial tension and obvious increase of interfacial pressure, thus laying a key theoretical foundation for its application as a natural emulsifying component in food colloid systems.

### 3.12 Interfacial adsorption kinetics

Typically, interfacial tension primarily serves as an indicator of the general tendency for surface-active compounds to accumulate at the interface between oil and water. In contrast, interfacial adsorption kinetics can deeply reveal the microscopic mechanisms such as diffusion migration, interfacial penetration, and molecular rearrangement experienced during the adsorption process. Consequently, the present research extended its systematic examination to assess how varying germination durations influence the kinetic behavior of BRPI during its adsorption at the oil-water interface. Fig. 3C/D respectively show the adsorption kinetic processes of BRPI at the oil-water interface under different germination times, where Fig. 3C represents the time-dependent diffusion adsorption process (*K*_*d*_) of protein molecules at the oil-water interface, and Fig. 3D represents the time-dependent penetration adsorption (*K*_*p*_) and rearrangement adsorption (*K*_*r*_) processes of protein molecules at the oil-water interface. However, the above images can only present the corresponding adsorption trends and lack quantitative analysis of different adsorption stages. Therefore, it is necessary to further perform linear fitting on the adsorption dynamics of BRPI during the diffusion, penetration, and rearrangement adsorption stages at the oil-water interface.

The results of linear fitting are presented in Table 1. Analysis of this table reveals notable variations in the diffusion (*K*_*d*_), penetration (*K*_*p*_), and rearrangement (*K*_*r*_) rates among the various samples at the oil-water interface. Specifically, during the stage of diffusion adsorption, the *K*_*d*_ value for sample S2 is considerably smaller compared to that of sample S1. This observation can likely be explained by the larger particle size and reduced solubility of sample S2, factors that hinder its diffusion toward the interface between oil and water. Interestingly, as the germination period was progressively extended from 8 to 24 h, the *K*_*d*_ of BRPI gradually increases, reaching the maximum value (0.0924) in the S5 sample after 24 h of germination, indicating that it has the strongest initial interfacial adsorption capability at the oil-water interface, protein molecules can migrate more rapidly from the bulk solution to the interface region, thereby effectively reducing interfacial tension (Fig. 3A) and enhancing interfacial pressure (Fig. 3B). This alteration can likely be explained by the smaller particle size and higher solubility of BRPI under this condition. Smaller protein molecules usually have faster diffusion rates, and better solubility means more uniform dispersion in the aqueous phase and easier access to the interface (Qu et al., 2021; Zhang et al., 2021).

**Table 1:** Effects of different germination times on the diffusion rate (*K*_*d*_), penetration rate (*K*_*p*_), and rearrangement rate (*K*_*r*_) of BRPI at the oil-water interface.

Samples	*K*_*d*_(mN/m^−1^·s^−0.5^)	*R*^*2*^	*K*_*p*_×10^3^(s^−1^)	*R*^*2*^	*K*_*r*_×10^3^(s^−1^)	*R*^*2*^
S1	0.0808 ± 0.0010^d^	0.991	2.07 ± 0.04^d^	0.996	4.51 ± 0.04^c^	0.990
S2	0.0710 ± 0.0008^e^	0.995	2.15 ± 0.01^d^	0.992	4.57 ± 0.03^c^	0.989
S3	0.0850 ± 0.0011^c^	0.989	2.83 ± 0.03^c^	0.989	4.68 ± 0.06^bc^	0.994
S4	0.0887 ± 0.0013^b^	0.996	2.96 ± 0.10^b^	0.993	4.83 ± 0.09^b^	0.994
S5	0.0924 ± 0.0018^a^	0.983	3.49 ± 0.10^a^	0.985	5.30 ± 0.29^a^	0.991

Further analysis revealed that the rearrangement rate constant (*K*_*r*_) was significantly higher than the penetration rate constant (*K*_*p*_) across all samples, suggesting that conformational rearrangement plays a more dominant role than initial penetration during the formation and stabilization of interfacial films (Niu et al., 2023; Tian et al., 2021). Interestingly, although the diffusion coefficient (*K*_*d*_) of S2 was significantly lower than that of S1, no statistically significant differences were observed in their *K*_*p*_ and *K*_*r*_ values (*p* > 0.05). This discrepancy is best understood by considering the distinct physical meanings of these parameters: *K*_*d*_ characterizes the diffusion kinetics of proteins from the bulk phase to the interface and is directly linked to the initial rate of interfacial pressure increase, whereas *K*_*p*_ and *K*_*r*_ are apparent rate constants derived from fitting the subsequent stages of the normalized interfacial pressure response curves, reflecting the penetration depth and conformational rearrangement efficiency of proteins already adsorbed at the interface, respectively. These three parameters correspond to different physical stages of the adsorption process. The larger particle size and reduced solubility of S2 are consistent with hindered diffusion toward the interface; however, during the subsequent kinetics of penetration and rearrangement at the oil-water interface, this sample retains conformational flexibility and surface hydrophobicity comparable to those of S1 (as shown in Fig. 2B and D), resulting in similar *K*_*p*_ and *K*_*r*_ values for both samples. Further investigation showed that extending germination time to 24 h (sample S5) resulted in a peak value of *K*_*r*_ for BRPI (5.30), which was significantly higher than that of other treated groups. This enhancement can be attributed to moderate hydrolysis induced by germination, which synergistically facilitates rapid anchoring and efficient rearrangement at the oil-water interface by increasing protein molecular flexibility and exposing additional hydrophobic residues (Y＀＀Y. Chen et al., 2023; Ma et al., 2024). In conclusion, germination treatment serves as a green and controllable physicochemical modification strategy that systematically optimizes the interfacial activity and multi-stage adsorption kinetics of BRPI, thereby providing both theoretical insights and technical support for the design of highly stable plant protein emulsions.

### 3.13 Interfacial dilatational rheology

While interfacial tension serves as a useful indicator for monitoring the dynamic adsorption process of surface-active molecules like proteins at the oil-water interface, offering an indirect measure of their emulsification capacity. Nonetheless, this metric does not capture the evolution of molecular adsorption conformations or the response of the protein membrane when subjected to interfacial mechanical deformations, such as shear or expansion. These interfacial mechanical responses in linear or nonlinear ranges are precisely the key to determining the long-term stability of metastable emulsions in multiphase systems (Wang et al., 2026a). Therefore, the dilatational rheological properties of BRPI treated with different germination times at the oil-water interface were further investigated in-depth. The interfacial viscoelastic properties (*E*), along with the elastic (*E*_*d*_) and viscous (*E*_*v*_) components for various BRPI samples, are depicted as a function of time in Fig. 4A, B, and 4C, respectively. It is obvious that with the extension of adsorption time, the curve distributions of *E*, *E*_*d*_, and *E*_*v*_ of all samples show a kinetic trend of first rising and then stabilizing, from relatively loose to gradually dense. This indicates that at the onset of adsorption, the number of protein molecules adsorbed at the oil-water interface is small, the protein coverage on the interface is low, the molecular arrangement is sparse, the interaction is weak, and the interfacial film is in the initial formation stage. With the further increase of adsorption time, a greater number of protein molecules are deposited at the interface, the molecular arrangement tends to be dense, and the interaction gradually increases, thereby improving the structural integrity and mechanical strength of the interfacial film. Further studies found that the *E*_*d*_ values of all samples are close to the *E* values and much larger than the *E*_*v*_ values, indicating that all samples adsorbed to the oil-water interface region results in the formation of a film dominated by elastic properties, which can effectively resist external disturbances and maintain the integrity of the interface, which is essential for ensuring the prolonged stability of the emulsion system (Ikenaga and Sagis, 2024).

**Fig. 4:**
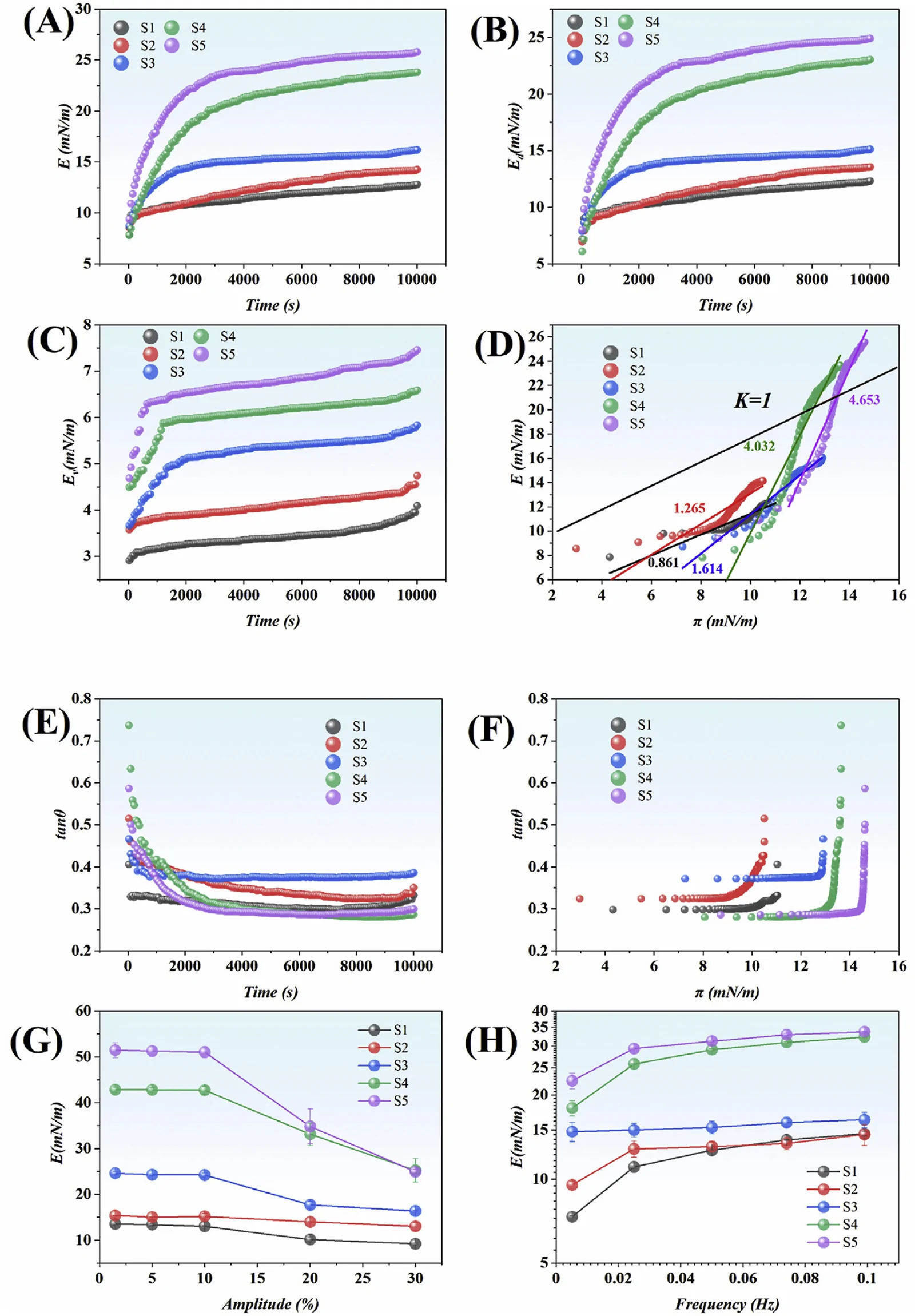

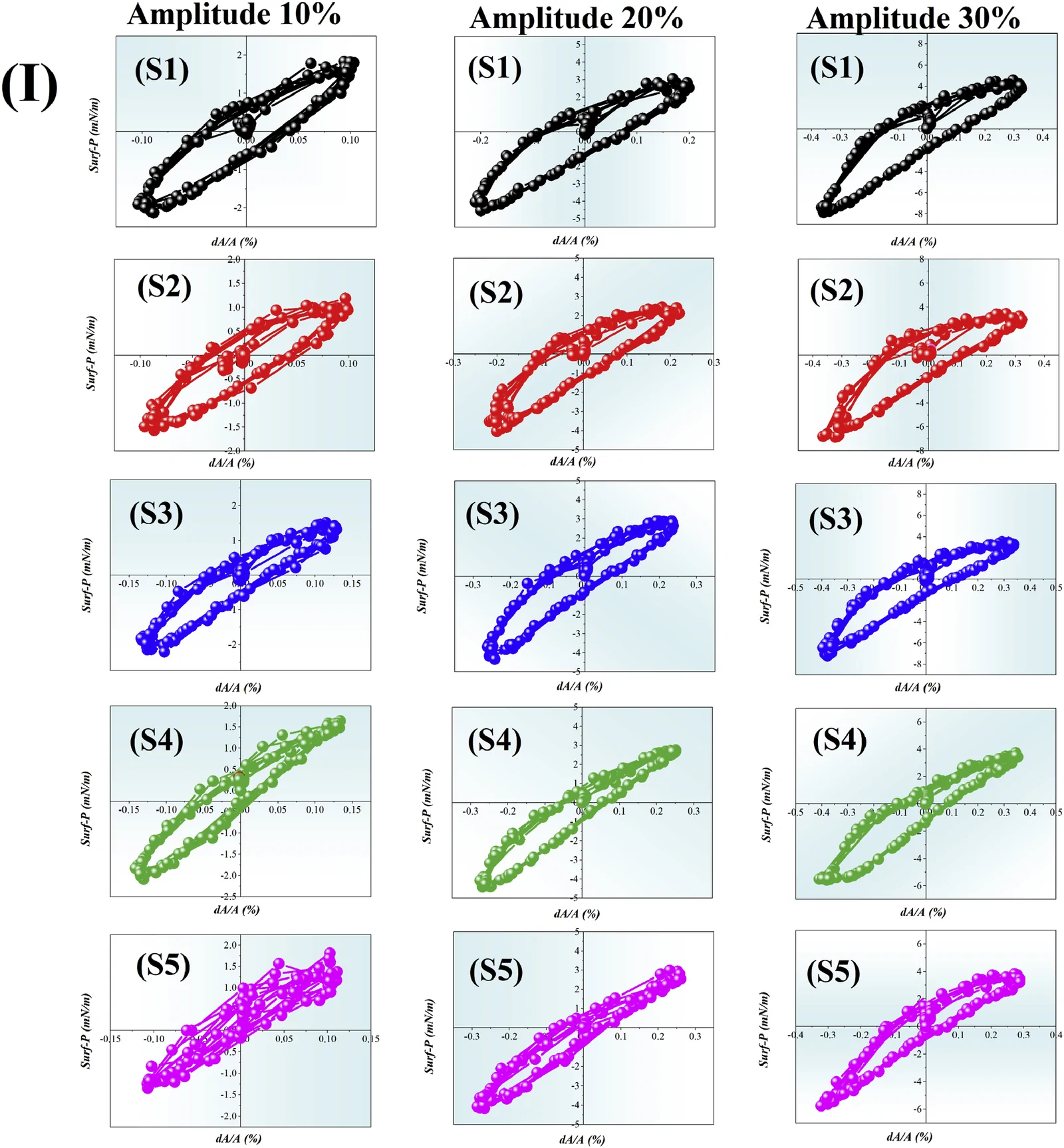
Effects of different germination times on the dilatational rheological properties of BRPI at the oil-water interface. Figures A, B, C, and D represent the time-dependent curves of the interfacial viscoelastic modulus (*E*), interfacial elastic modulus (*E*_*d*_), interfacial viscous modulus (*E*_*v*_), and the relationship between interfacial viscoelastic modulus (*E*) and interfacial pressure (*π*), respectively; Figures E, F, G, H, and I sequentially show the time-dependent tangent value (*tanθ*), the relationship between tangent value (*tanθ*) and interfacial pressure (*π*), the interfacial viscoelastic modulus (*E*) as a function of amplitude, the interfacial viscoelastic modulus (*E*) as a function of frequency, and the Lissajous figures at different amplitudes.

However, there are significant differences in the elastic interfacial films formed between different samples. Compared with the S1 sample, the *E*_*d*_ curve distribution of BRPI gradually increases with the gradual extension of germination time (0-24h), indicating that germination treatment can promote BRPI to form an elastic-dominated interfacial film at the oil-water interface region. Further research found that the S5 sample with a germination time of 24 h showed the highest *E*_*d*_ curve distribution, increasing from 7.993 mN/m to 24.888 mN/m, far exceeding other treatment samples, indicating that this sample formed a more elastic interfacial film at the oil-water interface. This improvement may be attributed to the higher conformational flexibility and surface hydrophobicity of BRPI following germination under this condition, which enables protein molecules to spread and rearrange faster and more effectively when adsorbed to the oil-water interface (Table 1), thus forming a tighter and more strongly interacting elastic interfacial film (Niu et al., 2023; Wang et al., 2026a).

Subsequently, linear fitting was performed on the *E–π* curves of various protein samples to obtain their slope values. Typically, under ideal adsorption conditions, the theoretical slope of the *E–π* curve is 1, indicating that adsorbed molecules exist in an independent, non-interacting gas-like state at the interface, where intermolecular forces are negligible (Liu, 2015). When the measured slope is significantly greater than 1, it reflects the presence of strong synergistic interactions among adsorbed molecules, including hydrogen bonds, van der Waals forces, hydrophobic interactions, and electrostatic interactions, leading to deviations from ideal adsorption behavior (Niu et al., 2023). Such strong interactions help construct interfacial films with dense structures and excellent mechanical strength, thereby effectively enhancing the long-term stability of metastable emulsion systems (Shi et al., 2024). Fig. 4D shows the *E–π* curve distributions of BRPI under different germination times. It is evident from the figure that the *E* values of all samples progressively rise as the *π* values increase, and the curves exhibit a trend of being loose initially and then dense. This indicates that the number of protein molecules adsorbed at the oil-water interface gradually increases, thereby enhancing the interactions between protein molecules adsorbed in the interface region, which is consistent with the thermodynamic framework of interfacial adsorption proposed by *Lucassen-Reynders* (Lucassen-Reynders et al., 1975). Further studies revealed significant differences in the *E–π* slopes among different samples. Specifically, compared with the S1 sample (slope = 0.861), the *E–π* slope of BRPI progressive rise as germination time is extended from 0 to 24 h, suggesting that germination treatment can enhance the interactions of BRPI adsorbed at the oil-water interface. Consequently, interfacial films with greater mechanical strength are formed, which helps in maintaining the stability of the resulting emulsion. When the germination time reaches 24 h, the *E–π* slope of the S5 sample reaches the highest peak (4.653), suggesting that under this condition, the BRPI have stronger interactions when adsorbed at the oil-water interface, forming an interfacial film with the greatest strength. These findings align with the results of interfacial pressure (Fig. 3B) and interfacial viscoelastic modulus (Fig. 4A), further confirming that germination treatment can not only significantly enhance the adsorption capacity of BRPI at the oil-water interface but also improve the structural integrity and mechanical stability of the subsequently formed interfacial films. In addition, the phase angle (*θ*) between dilatational strain and dilatational stress is a key parameter characterizing the viscoelastic behavior of adsorbed films, and its tangent value *tanθ* is defined as the ratio of *E*_*v*_ to *E*_*d*_ (Fig. 4E). Based on this, we constructed the *tanθ–π* curve distributions of BRPI. As shown in Fig. 4, the *tanθ–π* curves of all samples at the oil-water interface exhibit a similar trend: at higher *π* values, *tanθ* is lower (≤1), indicating that the interfacial films formed by BRPI at the oil-water interface are dominated by elasticity (Shi et al., 2024), which further verifies the results of *E*_*d*_ (Fig. 4B).

Furthermore, the interfacial viscoelastic modulus of BRPI molecules at the oil-water interface after adsorption equilibrium was investigated as a function of amplitude (1.5–30%) (Fig. 4G) or frequency (0.005–0.1 Hz) (Fig. 4H). From Fig. 4G, under small amplitude conditions (≤10%), the *E* values of all samples were independent of the applied amplitude, implying that this amplitude range falls within the linear viscoelastic response region (Zhan et al., 2019). Further studies showed that as the amplitude gradually increased (>10%), the *E* values of all samples exhibited an overall decreasing trend, indicating that the interfacial layer underwent a nonlinear response at larger amplitudes. This nonlinear behavior typically arises because, at large deformation amplitudes, the shear or dilatational forces acting on the interface may overcome intermolecular attractive forces, leading to disordered arrangement of surfactant molecules or even desorption of some molecules from the interface, thereby reducing the structural integrity and mechanical strength of the interface (Niu et al., 2023; Shen et al., 2023). Further research revealed significant differences in the interfacial viscoelastic modulus among different samples under the same amplitude variation conditions. Compared with the S1 sample, increasing the germination time could significantly improve the interfacial viscoelastic modulus of BRPI under the experimental amplitude conditions in this study, with the S5 sample (germination time of 24 h) showing the highest interfacial viscoelastic modulus. Notably, although this sample also exhibited a decreasing trend in interfacial viscoelastic modulus with increasing amplitude, which might lead to disordered arrangement at the oil-water interface or even desorption of some molecules, the BRPI molecules under this condition might have a higher adsorption amount at the oil-water interface and form a more viscoelastic interfacial film, which is verified by data from interfacial tension (Fig. 3A), interfacial pressure (Fig. 3B), and interfacial viscoelastic modulus (Fig. 4A). Therefore, the S5 sample still maintained a higher interfacial viscoelasticity under the experimental amplitudes.

Furthermore, the frequency-dependent viscoelastic properties of BRPI molecules at the oil-water interface were examined across a range of 0.005 to 0.1 Hz, following the attainment of adsorption equilibrium. As shown in Fig. 4H, the *E* of all samples exhibited a progressive rise as the frequency was increased. This frequency dependence of the modulus suggests that the dominant role of time-dependent effects such as rearrangement, adsorption, and relaxation mechanisms gradually strengthens (Rühs et al., 2013; Zhan et al., 2019). Further studies found that similar to the interfacial viscoelastic behavior under the same amplitude variation conditions, there were also marked differences in the interfacial viscoelasticity of different samples under the same frequency variation conditions. Specifically, compared with the S1 sample, the *E* of BRPI significantly increased with the extension of germination time (0–24 h). This result indicates that germination treatment can not only enhance its interfacial viscoelastic response within the amplitude range of 1.5%–30% but also improve its interfacial dynamic mechanical properties within the frequency range of 0.005–0.1 Hz, thereby synergistically enhancing its emulsification stability and tolerance to external mechanical disturbances in complex food systems. Further studies showed that the S5 sample with a germination time of 24 h exhibited the highest *E* response under this specific frequency condition, which further indicates that the BRPI under this germination condition can form a strong interfacial viscoelastic film when adsorbed from the bulk solution to the oil-water interface, thereby maintaining the stability of the emulsion.

Furthermore, this study also conducted an in-depth investigation into the nonlinear response characteristics of interfacial dilatational rheological behavior. In food systems, emulsions often experience mechanical stresses such as stirring, pumping, and shearing during production, transportation, and storage. These processes typically involve large deformations, pushing the interfacial film into the nonlinear rheological region (Wang et al., 2025). Therefore, relying solely on linear interfacial dilatational rheology cannot fully evaluate the stability of interfacial films under practical application conditions. In contrast, nonlinear dilatational rheology, especially when combined with Lissajous figure analysis, can more realistically reflect the response of protein adsorption layers under these intense conditions, thereby enabling a more comprehensive understanding and prediction of emulsion stability (Niu et al., 2023; Wang and Selomulya, 2022). Fig. 4I shows the Lissajous figures formed by BRPI with different germination times after reaching adsorption equilibrium at the oil-water interface, generated under three amplitude conditions (10%, 20%, and 30%). Specifically, at 10% amplitude, all samples exhibited elliptical Lissajous figures, indicating a sinusoidal relationship between stress and strain. This suggests that the BRPI at the oil-water interface under this amplitude condition display a linear viscoelastic response, with no irreversible changes to the interfacial film structure (De Groot et al., 2023). Interestingly, when the amplitude reached 20%, the Lissajous figures of all samples began to show asymmetry, marking a gradual deviation from linear viscoelastic response at the oil-water interface. This implies that under this amplitude condition, protein molecules at the interface start to form aggregates through strong interactions. The formation of these aggregates causes the interfacial film to gradually transition from a liquid-like to a more solid-like state, with changes in its mechanical strength and structural properties, and the stress-strain relationship may no longer follow a sinusoidal periodic response (Li et al., 2022; Yang et al., 2023; Zhang et al., 2022). Further research found that when the amplitude reached 30%, the Lissajous figures of all samples exhibited asymmetric behavior: strain hardening during compression and strain softening during stretching. Similar findings have been reported for mung bean protein concentrate, which is a typical characteristic of proteins at interfaces (Kontogiorgos and Prakash, 2023). This suggests that the viscoelastic network formed by BRPI at the oil-water interface undergoes structural disruption during stretching; during compression, as the aggregation density of proteins at the oil-water interface increases and intermolecular interactions strengthen, the interface tends to approach a jammed state (Sagis and Fischer, 2014; Yang et al., 2022). These results are aligned with the *E* of BRPI molecules at the oil-water interface after adsorption equilibrium as a function of amplitude (1.5–30%) (Fig. 4G).

Further investigation revealed that, compared with sample S1, the Lissajous figures of BRPI-stabilized interfaces gradually narrowed as germination time increased from 0 to 24 h, which narrowing reflects reduced energy dissipation during deformation, indicating an accelerated elastic response and enhanced interfacial elasticity induced by germination (Li et al., 2026). This improvement may be attributed to the enhanced surface hydrophobicity and conformational flexibility of germinated BRPI (as shown in Fig. 2D and B). Surface hydrophobicity serves as the primary thermodynamic driving force for protein migration from the aqueous phase to the oil-water interface; higher hydrophobicity strengthens the affinity between proteins and the non-polar oil phase, thereby significantly lowering the adsorption energy barrier and increasing the adsorption rate (Wang et al., 2026a). Beyond this thermodynamically driven acceleration of adsorption, improved conformational flexibility is a key kinetic factor determining the quality of the interfacial film. Rigid globular proteins often struggle to undergo substantial structural unfolding upon adsorbing to the interface, resulting in limited coverage area and forming loose interfacial films with insufficient mechanical strength (Tang and Shen, 2015). In contrast, germinated BRPI exhibits greater conformational flexibility due to a tendency toward disordered secondary structure (Fig. 2A). This flexibility enables protein molecules to rapidly undergo partial unfolding once they reach the oil-water interface, rearranging themselves to maximize their contact area with the interface (Yang et al., 2023). Such unfolding and rearrangement not only fill gaps at the interface but also promote side-chain interactions between adjacent protein molecules (such as hydrophobic interactions, hydrogen bonding, and disulfide exchange), leading to the formation of a dense, elasticity-dominated network structure (Wang et al., 2026b). Furthermore, the partial hydrolysis of BRPI induced by enhanced protease activity during germination (Fig. 1B/E and 2F) promotes the exposure of surface charges, thereby strengthening intermolecular electrostatic repulsion and reducing aggregate size. These changes generally facilitate the rapid adsorption of more BRPI molecules at the oil-water interface (Wang et al., 2026a). Subsequently, it was found that when the germination time reached 24 h, the Lissajous figure of the BRPI sample was the narrowest, indicating that the interfacial film formed under these conditions possessed stronger elasticity and could better withstand large deformation disturbances from the external environment (Yang et al., 2024). This further corroborates the results obtained from the dynamic interfacial dilatational modulus (*E*_*d*_) measurements (see Fig. 4B).

In summary, BRPI after germination treatment, especially those germinated for 24 h, can form a denser, more strongly interacting, and elasticity-dominated interfacial viscoelastic film at the oil-water interface. This is reflected in their higher *E*, *E–π* slope significantly greater than 1, and lower *tanθ* value. Additionally, even under large-amplitude disturbances, germination-treated BRPI can still maintain strong interfacial viscoelasticity, which is crucial for stabilizing multiphase emulsion systems.

### 3.14 Microstructure, emulsifying activity and emulsion stability of emulsion

Typically, the stability of an emulsion is closely related to its structural size: larger emulsion droplet sizes tend to lead to stratification and phase separation, whereas smaller sizes correspond to better emulsion stability (Wang et al., 2026a). Fig. 5A illustrates the effect of different BRPI samples used as emulsion stabilizers on the structural size of the emulsion. Clearly, at the initial stage of emulsion formation (0 min), the emulsion stabilized by the S1 sample exhibited a significantly larger microstructure, indicating relatively poor stability and difficulty in resisting external strain disturbances. Further studies showed that as germination time gradually extended (0–24 h), the structural size of the emulsion stabilized by BRPI gradually decreased, and the size distribution became more uniform. The S5 sample (24 h germination) displayed smaller and more uniformly distributed emulsion droplets compared to other samples, a trend that was also observed at 60 min after emulsion formation. This can be explained by the lower interfacial tension (Fig. 3A) and higher interfacial elastic modulus (Fig. 4B) of the BRPI under this germination condition relative to other samples. Previous studies have shown that emulsion droplet size is closely linked to the ability of protein molecules to reduce interfacial tension at the oil-water interface and form elastic interfacial films: lower interfacial tension means less energy is required for emulsion formation, which favors the production of smaller, more uniform droplets under the same shearing conditions (Jia et al., 2022; Ravera et al., 2021). Additionally, the mechanical properties of the interfacial film formed by protein molecules at the oil-water interface are critical, with the interfacial elastic modulus serving as a key indicator of the film's ability to resist compression and shear deformation. A highly elastic interfacial film can effectively provide steric hindrance and a mechanical barrier during droplet collisions, preventing droplet coalescence and thus delaying emulsion flocculation and stratification (Nooshkam et al., 2023; Ravera et al., 2021).

**Fig. 5:**
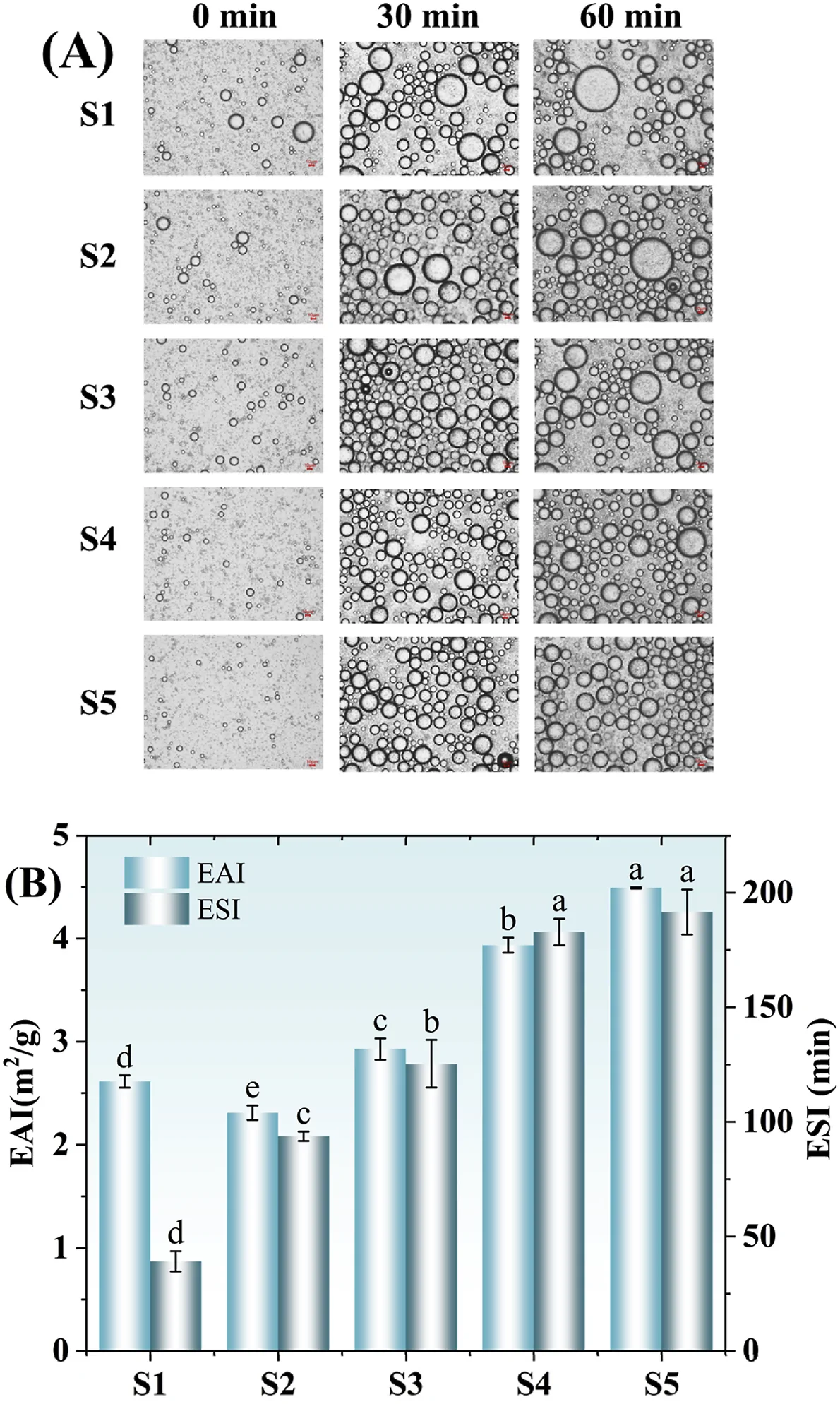
Effects of different germination times on the microstructure (A), emulsifying activity index (EAI), and emulsion stability index (ESI) (B) of emulsions stabilized by BRPI.

Beyond microstructure, the emulsifying activity and emulsion stability of protein molecules can also be quantitatively characterized by the emulsifying activity index (EAI) and emulsifying stability index (ESI). As shown in Fig. 5B, there were significant differences in EAI and ESI among different samples. In terms of EAI, compared to the S1 sample (2.61 m^2^/g), the EAI of the S2 sample decreased significantly (2.31 m^2^/g). However, as germination time further extended (8–24 h), the emulsifying activity of BRPI gradually improved, with the S5 sample exhibiting the highest EAI (4.49 m^2^/g) among all samples. This result is aligned with the conclusions from interfacial tension and diffusion adsorption rate analyses (Fig. 3A/Table 1). Previous studies have indicated that EAI is primarily determined by interfacial tension and adsorption rate, these factors are crucial during the process of emulsion formation (Wang et al., 2026a). Interestingly, the trend of ESI slightly differed from that of EAI. Specifically, compared to the S1 sample (39.15 min), the ESI of BRPI gradually improved with the extension of germination time (0–24 h), peaking at 191.49 min in the S5 sample. This indicates that the BRPI under this germination condition has higher emulsion stability, which is also verified by the microstructure of the emulsion it stabilized (Fig. 5A). The mechanism can be attributed to the faster penetration and rearrangement capabilities of the S5 sample at the oil-water interface (Table 1), which help form a dense, highly cross-linked interfacial film with excellent elasticity (Fig. 4B), thereby significantly enhancing its resistance to complex external stresses and maintaining emulsion stability (Wang et al., 2025). In summary, germination treatment effectively improves emulsion stability by optimizing the interfacial activity and interfacial film mechanical properties of BRPI.

### 3.15 Correlation analysis and potential mechanism

To systematically clarify the mechanism underlying the effects of germination treatment on the structural properties, interfacial adsorption behavior, and emulsifying performance of BRPI, this study employed Pearson correlation model to quantitatively evaluate the statistical associations between structural parameters, interfacial adsorption kinetic parameters (*K*_*d*_, *K*_*p*_, *K*_*r*_), interfacial film elasticity parameters (*E*_*d*_, *E-π* slope, *tanθ*), and emulsifying performance indices (EAI, ESI). As shown in Fig. 6A: (I) The average particle size exhibited extremely significant negative correlations with *K*_*d*_, *K*_*p*_, *K*_*r*_, *E*_*d*_, and the *E-π* slope (*p* < 0.01), indicating that a reduction in particle size can significantly enhance the adsorption rate of protein molecules at the oil–water interface and the capacity for elastic interfacial film formation. (II) The average particle size showed a significant positive correlation with *tanθ* (*p* < 0.05), suggesting that smaller particle sizes favor the enhancement of the elasticity-dominant characteristics of the interfacial film. (III) The zeta potential displayed extremely significant positive correlations with *K*_*d*_, *K*_*p*_, *K*_*r*_, *E*_*d*_, and the *E-π* slope (*p* < 0.01), reflecting that stronger electrostatic repulsion helps maintain protein dispersion stability, reduce aggregation tendency, and thus promote efficient interfacial adsorption and elastic network construction. (IV) Surface hydrophobicity demonstrated extremely significant positive correlations with *K*_*d*_, *K*_*p*_, *E*_*d*_, and the *E-π* slope (*p* < 0.01), and a significant positive correlation with *K*_*r*_ (*p* < 0.05), indicating that moderately enhanced surface hydrophobicity can effectively drive the directional anchoring of proteins at the oil-water interface and support the assembly of elastic interfacial films. (V) Wettability showed extremely significant positive correlations with all interfacial parameters (*K*_*d*_, *K*_*p*_, *K*_*r*_, *E*_*d*_, and the *E-π* slope) (*p* < 0.01), confirming that it can serve as a key physicochemical indicator for characterizing protein interfacial affinity and adsorption efficiency. (VI) Conformational flexibility exhibited extremely significant positive correlations with *K*_*d*_, *K*_*p*_, *E*_*d*_, and the *E-π* slope (*p* < 0.01), and a significant positive correlation with *K*_*r*_ (*p* < 0.05), suggesting that higher conformational dynamics facilitate the rapid rearrangement of proteins at the interface and the formation of three-dimensional networks. (VII) The *β*-sheet content displayed extremely significant negative correlations with all interfacial parameters (*K*_*d*_, *K*_*p*_, *K*_*r*_, *E*_*d*_, and the *E-π* slope) (*p* < 0.01), implying that its structural rigidity may limit the interfacial adaptability of molecules. (VIII) The *β*-turn content showed extremely significant positive correlations with *K*_*d*_, *K*_*p*_, *K*_*r*_, *E*_*d*_, and the *E-π* slope (p < 0.01), but a significant negative correlation with *tanθ* (*p* < 0.05), indicating that an increase in *β*-turns not only promotes interfacial anchoring and elastic film construction but also tends to reduce the viscous contribution of the interface, reinforcing the elasticity-dominant mechanical response. (IX) Solubility exhibited extremely significant positive correlations with all interfacial parameters (*p* < 0.01), highlighting its fundamental role as a prerequisite for interfacial activity. (X) Turbidity demonstrated extremely significant negative correlations with *K*_*d*_, *K*_*p*_, *E*_*d*_, and the *E-π* slope (*p* < 0.01), and a significant negative correlation with *K*_*r*_ (*p* < 0.05), indicating that increased solution turbidity often reflects intensified protein aggregation, thereby impairing interfacial adsorption efficiency and film structural integrity. (XI) The emulsifying activity index (EAI) showed extremely significant positive correlations with *K*_*d*_, *K*_*p*_, *K*_*r*_, *E*_*d*_, and the *E-π* slope (*p* < 0.01), but an extremely significant negative correlation with *tanθ* (*p* < 0.01), suggesting that high interfacial adsorption efficiency and a strong elastic interfacial film jointly determine EAI performance, while a lower *tanθ* value (i.e., higher elasticity proportion) further optimizes emulsifying capacity. (XII) The emulsifying stability index (ESI) also displayed extremely significant positive correlations with all the aforementioned interfacial parameters (*p* < 0.01), confirming that the structural robustness of the interfacial film is the core foundation of long-term anti-coalescence ability.

**Fig. 6:**
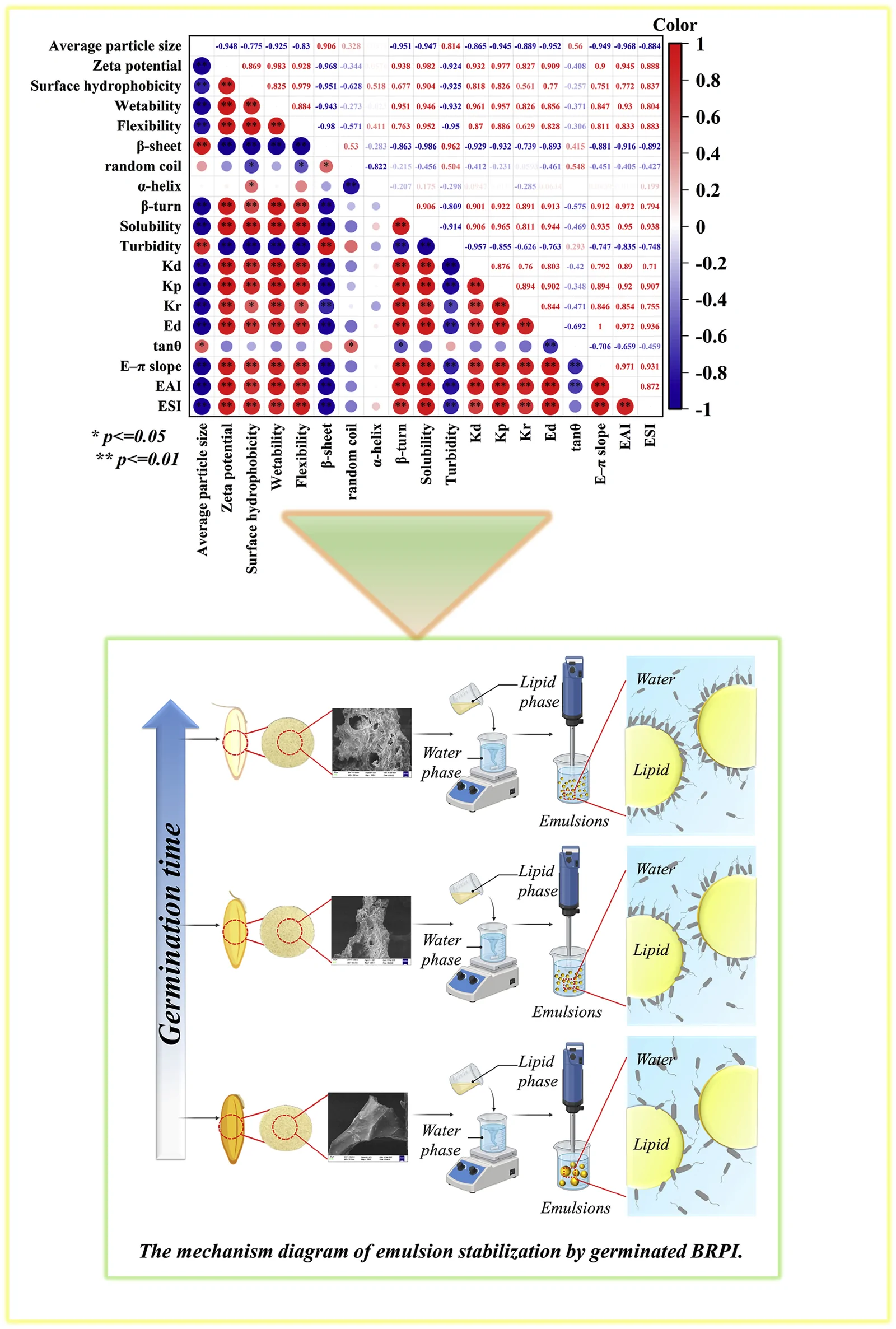
Pearson correlation analysis between the structural properties, interfacial behavior, emulsifying properties of germinated BRPI (A), and its mechanism model (B).

In summary, the results of the correlation analysis are highly consistent with the previously discussed structural-interface-functional relationships, providing robust statistical support for the mechanistic interpretation.

Based on the above findings, a mechanism model linking structural characteristics, interfacial adsorption, and emulsion stability during BRPI germination was further constructed (Fig. 6B). As illustrated, non-germinated BRPI (samples S1 and S2) exhibits a dense and compact molecular conformation, leading to limited exposure of charged groups and hydrophobic residues, a significant increase in particle hydrated size, and subsequent inhibition of conformational flexibility and aqueous solubility, ultimately impairing interfacial adsorption kinetics and adsorption capacity. In contrast, with prolonged germination, the molecular structure of BRPI undergoes progressive relaxation, forming a more open and porous conformational state. This structural transition significantly enhances intermolecular electrostatic repulsion, leading to a marked reduction in hydrated particle size. Critically, germination treatment simultaneously improves the solubility, conformational flexibility, and surface hydrophobicity of BRPI. The synergistic effect of these structural properties not only promotes the rapid migration and anchoring of more protein molecules at the oil–water interface but also accelerates the penetration, rearrangement, and elastic network construction of the interfacial film in the late adsorption stage. Ultimately, a dense viscoelastic interfacial layer with high mechanical strength and anti-coalescence ability is formed, thereby endowing the emulsion with excellent long-term stability.

## 4 Conclusion

This study demonstrated that germination improved the emulsifying performance of BRPI through coordinated changes in protein structure, physicochemical properties, and interfacial behavior. Unlike soaking alone, which induced slight aggregation and decreased EAI, prolonged germination promoted partial protein hydrolysis and conformational loosening, accompanied by smaller particle sizes, greater absolute zeta potential, and increased solubility, conformational flexibility, and surface hydrophobicity. These changes were associated with more efficient interfacial adsorption and the formation of stronger, elasticity-dominated interfacial films. Among the investigated samples, BRPI germinated for 24 h exhibited the highest EAI and ESI, reaching 4.49 m^2^/g and 191.49 min, respectively, compared with 2.61 m^2^/g and 39.15 min for untreated BRPI.

Correlation analysis further linked these structural and interfacial changes to emulsifying performance. Smaller particle sizes and lower turbidity, together with higher solubility, flexibility, and surface hydrophobicity, were associated with faster interfacial adsorption and greater film elasticity. Both EAI and ESI were positively correlated with the diffusion, penetration, and rearrangement rate constants, the interfacial dilatational elastic modulus, and the *E–π* slope (*p* < 0.01), while EAI was negatively correlated with *tanθ* (*p* < 0.01). These associations support an integrated interpretation in which efficient adsorption facilitates the formation and coverage of newly generated droplets, whereas elastic interfacial films contribute to their subsequent stabilization. Thus, the functional benefits of germination reflect the combined contributions of protein dispersibility, conformational adaptability, and interfacial film mechanics. These findings support controlled germination as a promising strategy for developing BRPI-based emulsifiers.

Nevertheless, several potential processing challenges and limitations must be considered for its practical application in target food systems such as bakery products and plant-based milk beverages. First, germination not only induces the synthesis of proteases but may also generate reducing sugars and free amino acids, which are prone to triggering Maillard reactions during processing, leading to browning and alterations in flavor compounds. Second, microbiological safety risks associated with the high-humidity environment required for germination warrant attention. Furthermore, precise control of germination conditions remains challenging, potentially resulting in batch-to-batch variations in product quality. To address these issues, future strategies could involve integrating biological processing techniques (e.g., combining fermentation with germination), employing non-thermal physical field sterilization methods (such as radiofrequency treatment), and implementing sensor-based real-time monitoring systems for temperature and humidity.

## CRediT authorship contribution statement

**Zhuang-Peng Wang**: Conceptualization, Formal analysis, Resources, Writing the original draft. **Xin-wen Huang**: Software, Validation and Resources. **Yu Zhao**: Resources, Software, Project administration and Validation. **Hai-ping Liang and Kun Wang**: Data curation, Investigation and Supervision. **Lei-Yan Wu**: writing, review and editing, Funding acquisition.

## Date availability

Data will be made available on request.

## Declaration of competing interest

The authors declare that they have no known competing financial interests or personal relationships that could have appeared to influence the work reported in this paper.
