# Supplementary material for: Dynamic adsorption behavior and film-forming stabilization mechanism of brown rice protein isolate at the oil-water interface: Effects of short-term germination treatment
Source: Curr Res Food Sci. 2026 Nov 2;13:101615. doi: 10.1016/j.crfs.2026.101615 (PMC13756527; doi:10.1016/j.crfs.2026.101615)
Supplement: Supplementary file 1 [file mmc1.docx]

**Supplementary material**

Figure. S1 Distribution of amide I band spectra of BRPI at different germination times
